# The evolution of vitamin C biosynthesis and transport in animals

**DOI:** 10.1186/s12862-022-02040-7

**Published:** 2022-06-25

**Authors:** Pedro Duque, Cristina P. Vieira, Bárbara Bastos, Jorge Vieira

**Affiliations:** 1 Instituto de Ciências Biomédicas Abel Salazar (ICBAS), Universidade do Porto, Rua de Jorge Viterbo Ferreira, 228, 4050-313 Porto, Portugal; 2 Faculdade de Ciências da Universidade do Porto (FCUP), Rua do Campo Alegre, s/n, 4169-007 Porto, Portugal; 3 Instituto de Investigação e Inovação em Saúde (I3S), Universidade do Porto, Rua Alfredo Allen, 208, 4200-135 Porto, Portugal; 4 Instituto de Biologia Molecular e Celular (IBMC), Rua Alfredo Allen, 208, 4200-135 Porto, Portugal

**Keywords:** Ascorbic acid, GULO, Regucalcin, SVCT, Animals

## Abstract

**Background:**

Vitamin C (VC) is an indispensable antioxidant and co-factor for optimal function and development of eukaryotic cells. In animals, VC can be synthesized by the organism, acquired through the diet, or both. In the single VC synthesis pathway described in animals, the penultimate step is catalysed by Regucalcin, and the last step by l-gulonolactone oxidase (GULO). The *GULO* gene has been implicated in VC synthesis only, while *Regucalcin* has been shown to have multiple functions in mammals.

**Results:**

Both *GULO* and *Regucalcin* can be found in non-bilaterian, protostome and deuterostome species. *Regucalcin*, as here shown, is involved in multiple functions such as VC synthesis, calcium homeostasis, and the oxidative stress response in both Deuterostomes and Protostomes, and in insects in receptor-mediated uptake of hexamerin storage proteins from haemolymph. In Insecta and Nematoda, however, there is no *GULO* gene, and in the latter no *Regucalcin* gene, but species from these lineages are still able to synthesize VC, implying at least one novel synthesis pathway. In vertebrates, *SVCT1*, a gene that belongs to a family with up to five members, as here shown, is the only gene involved in the uptake of VC in the gut. This specificity is likely the result of a subfunctionalization event that happened at the base of the Craniata subphylum. *SVCT*-like genes present in non-Vertebrate animals are likely involved in both VC and nucleobase transport. It is also shown that in lineages where *GULO* has been lost, *SVCT1* is now an essential gene, while in lineages where *SVCT1* gene has been lost, *GULO* is now an essential gene.

**Conclusions:**

The simultaneous study, for the first time, of *GULO*, *Regucalcin* and *SVCT*s evolution provides a clear picture of VC synthesis/acquisition and reveals very different selective pressures in different animal taxonomic groups.

**Supplementary Information:**

The online version contains supplementary material available at 10.1186/s12862-022-02040-7.

## Background

In eukaryotes, Vitamin C (VC), also known as l-ascorbic acid or ascorbate, is an essential micronutrient for normal cell function, growth and development, acting as an antioxidant capable of detoxifying exogenous radical species or those generated during mitochondrial metabolism [1, 2]. VC is required for normal neuromodulation [3], protection against lipid peroxidation [4], and collagen biosynthesis, where it acts as a cofactor for collagen stabilization enzymes, namely prolyl and lysyl hydroxylases [5]. Elevated VC levels have been associated with protection against degenerative diseases and cancer, with several reports suggesting beneficial effects in cancer treatment and chemotherapy recovery [6]. Nevertheless, at high concentrations, and in the presence of catalytic metal ions such as ferric iron (Fe^3+^), VC can also have a pro-oxidative behavior through an indirect contribution in the Fenton reaction [6]. The radical species that are generated can act as triggers for lipid peroxidation [7], a self-proliferating chain reaction that ultimately leads to damage in membranes, proteins and DNA [8]. In humans, lack of VC leads to scurvy [9].

**Fig. 1:**
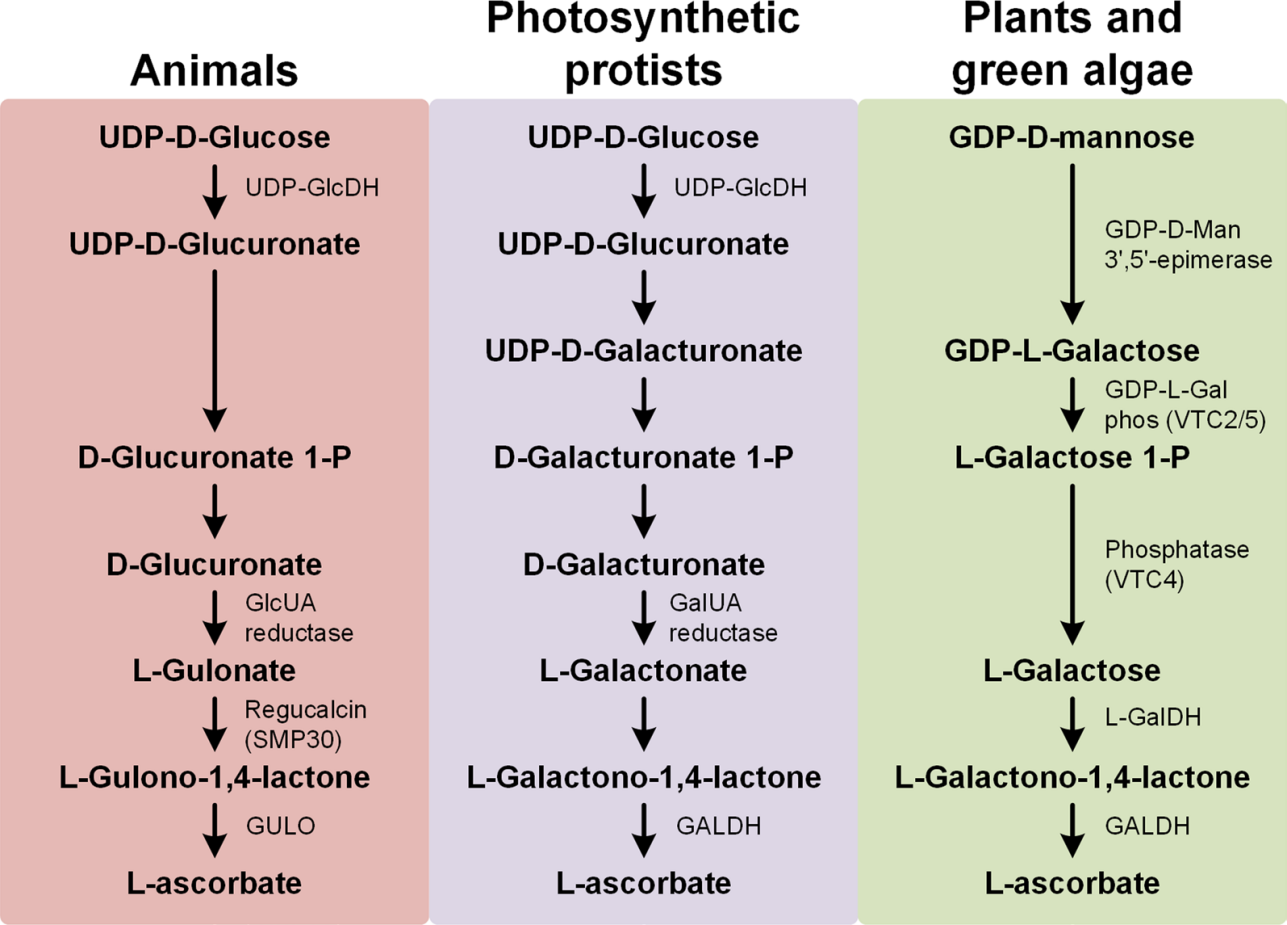
Graphic display of the currently known VC synthesis pathways. The final oxidation step of the distinct aldono-1,4-lactone to ascorbate is performed by a FAD-linked oxidase or dehydrogenase (GULO or GALDH). Photosynthetic protists appear to possess enzymatic components from animal and plant pathways, and due to this characteristic, the current described pathway for these species likely evolved from a secondary endosymbiosis event regarding a non-photosynthetic ancestor and algae [15]. The figure here presented and the corresponding description were adapted from the literature [14]

Being an essential vitamin, it is not surprising that VC is endogenously synthetized by a wide variety of species, ranging from commensal and parasitic bacteria [10–12] to highly complex eukaryotes, such as plants and animals [13–15]. Three main VC biosynthesis pathways have been described (Fig. 1), namely the photosynthetic protists, plant/green algae, and animal pathways [14]. In animals, d-glucose is used as the initial precursor, and after several enzymatic reactions is converted to l-gulonolactone by Regucalcin (in deuterostomians it is also known as Senescence marker protein 30 (SMP30)). l-gulonolactone then participates in a final oxidation reaction catalyzed by l-gulonolactone oxidase (GULO), leading to the formation of 2-keto-l-gulonolactone, which spontaneously enolizes to l-ascorbic acid [16, 17]. Not all animals are, however, capable of synthesizing VC. Within deuterostomes, humans (*Homo sapiens*), non-human primates, the guinea pig (*Cavia porcellus*), numerous bats, various birds, and the teleost fish have lost this ability, due to the complete or partial loss of the *GULO* gene [13]. Although *GULO* is also present in non-bilateria species, it is clear that this gene was lost in several protostomian lineages, such as insects and nematodes [15]. Since only one VC synthesis pathway has been described in animals, species belonging to these lineages were thought not to be able to synthesize VC. Nevertheless, at present, there are two insect species, namely *Drosophila melanogaster* (diptera), and *Bombyx mori* (lepidoptera), as well as one nematode (*Caenorhabditis elegans*), that have been shown to synthesize VC [18–20]. It should be noted that distantly related insect groups, such as the Diptera and the Lepidoptera, may have independently acquired de novo the ability to synthesize VC. Indeed, there is evidence that the ability to synthesize VC without the intervention of *GULO* may have arisen independently multiple times in protostomian evolution, since this gene is absent in nematodes and insects, but present in Arachnida species that are closer to insects [19, 21].

In *B. mori*, it has been suggested that the VC synthesis pathway could be identical to that of mammals and other vertebrates with the exception of the enzyme involved in the last step of the pathway [20]. Indeed, GULO-like activity has been detected in *B. mori*, although at reduced levels compared to other species [20]. Surprisingly, the protein identified as having GULO-like activity is a sterol reductase, in contrast to GULO that is an aldonolactone oxireductase [20, 22]. Since aldonolactone oxireductases and sterol reductases are distantly related flavoproteins [22], the ability of the *B. mori* sterol reductase to catalyze the oxidation of l-gulonolactone was unexpected.

In deuterostomians, apart from the substrate conversion role in the VC synthesis pathway, Regucalcin is also implicated in several physiological mechanisms, such as intracellular Ca^2+^ homeostasis, proteolysis, and signaling and oxidative stress regulation [23]. Despite the many functional roles played by this protein in deuterostomians, homozygous *Regucalcin* knockout mice are viable [24, 25]. It should be noted that there are no *Regucalcin* paralogs in *Mus musculus* that could compensate *Regucalcin*’s functions in homozygous knockout mice. Nevertheless, although viable, knockout mice still show increased cell senescence in several tissues and shorter life spans [26, 27].

In protostomians, the role of *Regucalcin* is much less clear, but in the dipteran *Sarcophaga peregrina*, the Anterior fat body protein encoded by the *AFP* gene (an homolog of *Regucalcin*) is almost exclusively found in the anterior fat body, and does not exhibit strong affinity towards calcium [28]. AFP may participate in the receptor-mediated uptake of hexamerin storage proteins from insect haemolymph by fat body cells, a mechanism that is only found in the Insecta. In another dipteran, *Calliphora vicina*, there is an *AFP* orthologue that encodes a protein that interacts with an hexamerin receptor [29], and in *D. melanogaster* Regucalcin has been found in the haemolymph [30], findings that strengthen this putative function. Therefore, it has been assumed that *Regucalcin* does not have a role in calcium homeostasis in *D. melanogaster* [31]. Nevertheless, in this species, there is a *Regucalcin* paralog, named *Drosophila cold acclimation* (*Dca*), that is restricted to the subgenus Sophophora [31, 32]. *Dca* shows high sequence identity with *Regucalcin* [31], and thus, could have similar functions. Nevertheless, Dca is currently implicated in the response to cold exposure and wing size variation in flies [25, 31–33], functions that are not easily reconciled with the role of a protein involved in the uptake of storage proteins by fat body cells. In the lineage leading to *Dca*, the nonsynonymous fixation rate is, however, increased, suggesting that *Dca* could have acquired a new function (neofunctionalization) after the duplication event [31]. The expression pattern of these genes is indeed distinct, since *Regucalcin* has a much higher expression level than *Dca* under normal conditions (information available at https://flybase.org/). Moreover, *Dca* is primarily expressed in the adult digestive system tissues and *Regucalcin* in the larval fat body and pupa [31, 34, 35]. There is no data on whether *D. melanogaster Regucalcin* is an essential gene, but *Dca* knockout flies have been shown to be viable [25].

In vertebrates, VC is primarily synthesized in either the liver or the kidney depending on the species being considered [13], but once synthesized, VC must be transported to the cells where it is needed. This process is mainly reliant on Sodium-dependent Vitamin C Transporters (SVCTs) and Sodium-independent facilitative glucose transporters (GLUTs) [6, 36, 37]. The contribution of GLUTs to the maintenance of favorable VC concentrations in cells is, however, small when compared to SVCTs, which are considered the main regulator of VC uptake [6, 38–40]. In vertebrates, SVCTs are a family of surface glycoproteins with four members (SVCT1 to 4), but only SVCT1 and SVCT2 proteins have been implicated in VC homeostasis [36]. These two proteins share a unique amino acid motif in transmembrane domain 10 (SSSP) that may be responsible for the substrate specificity [41]. SVCT1 is essentially expressed in the epithelial membranes of various organs, such as the intestine, kidney and liver, while SVCT2 has a generalized presence throughout the body [36, 42]. Therefore, SVCT1 protein has been implicated in specific VC uptake, whereas the SVCT2 transporter has been implicated in localized cell responses to oxidative stress [36, 40, 43]. No function has yet been attributed to *SVCT3*, but it does not seem to have VC or nucleobase affinity [36]. *SVCT4* is known to transport various nucleobases but not VC [44].

*SVCT1* and *SVCT2* genes are probably the result of a duplication event that preceded the divergence of bony fish (Osteichthyes) and tetrapods [37, 41]. The evolutionary history of the four members of the SVCT family is, however, not yet clear. Some authors consider *SVCT3* and *SVCT4* orphan genes [37, 42], while others indicate that *SVCT3* may have diverged early in evolution from *SVCT1* and *SVCT2* [41]. The evolutionary history of SVCTs in non-bilaterian and protostomians is also largely unknown. It has been, however, hypothesized that *SVCT4* is most similar to the *SVCT*s observed in these basal taxonomic groups [41]. Since the vertebrate *SVCT4* is not a VC transporter, if true, it could be that there are no VC transporters in non-bilaterian and protostomian, species, or that the vertebrate *SVCT4* lost its ancient VC transporter activity. The former hypothesis is highly unlikely. For instance, although the lepidopteran *B. mori* is able to synthesize VC, it does not synthesize enough of it during larval stages 3-LE up to 5-LE for its needs, and thus must rely on an external source of this nutrient [20]. Therefore, *B. mori* must have a functional VC transporter.

Using a combination of phylogenetic and protein structure analyses, as well as inferences on positively selected amino acid sites, the evolutionary history of *Regucalcin*-like and *SVCT*-like genes in animals is here clarified. These results are interpreted in the light of the recently published *GULO* phylogenetic and functional analyses [18–21], revealing a history of pseudogenization, neofunctionalization and sub-functionalization. The joint evolutionary history of *Regucalcin*, *GULO* and *SVCT*s, also explains why some animal lineages lost the ability to synthesize VC while other basal lineages did not.

## Results

### Contrasting patterns of *Regucalcin* gene duplication and loss in different animal lineages

*Regucalcin* presence/loss was addressed using restrictive criteria. Indeed, within a given taxonomic group, a minimum of three species showing no evidence for the presence of a gene was required to infer gene loss in that lineage, since most genomes do not have a 100% coverage.

**Fig. 2:**
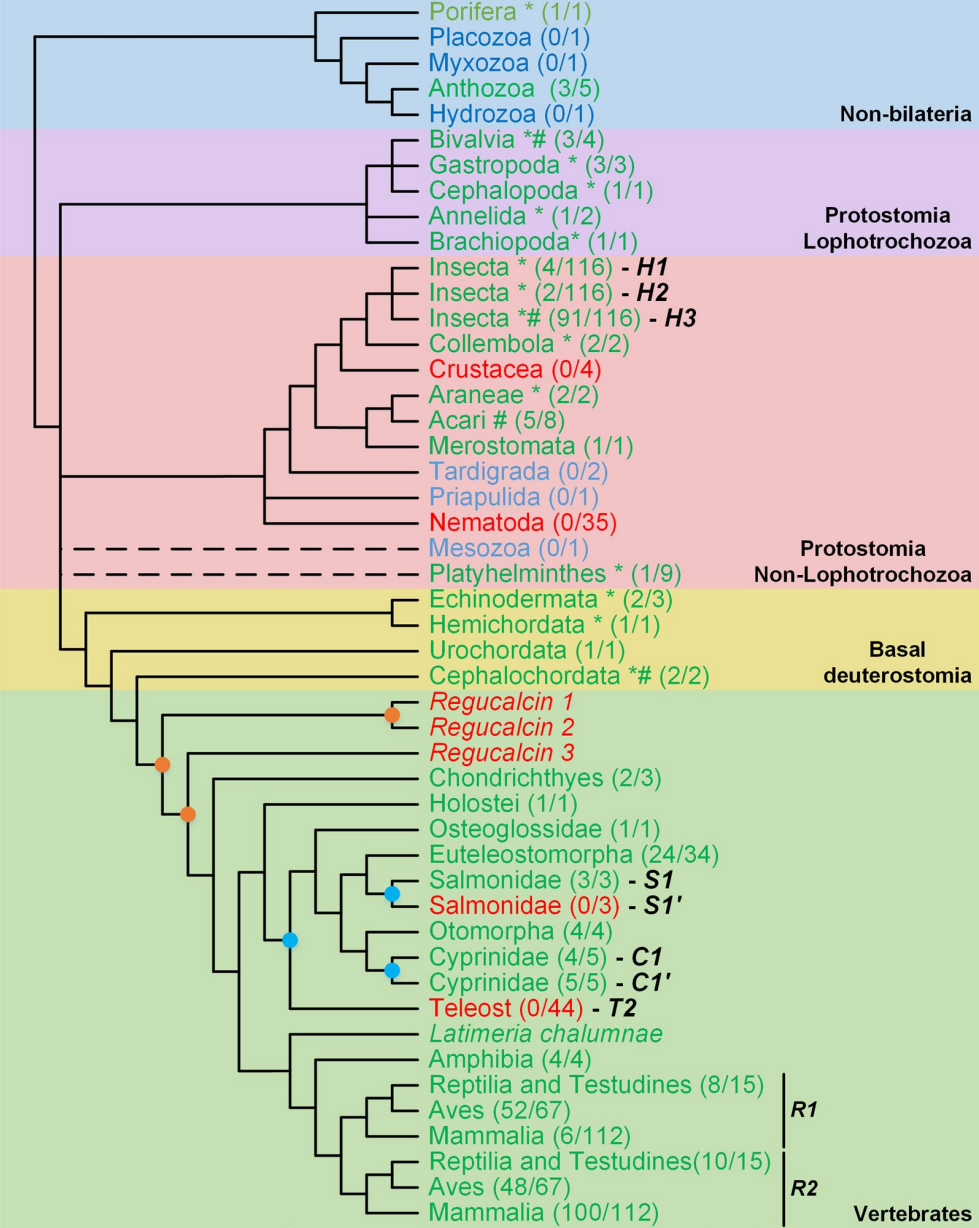
*Regucalcin* evolution across the animal kingdom. *Regucalcin* presence is highlighted in green, absence in red and uncertainty regarding gene loss/presence in light blue. Gene duplications affecting a single species of a genus are marked with a *, while those which affected two or more species from the same genus are marked with a #. The number of species present in the final dataset and total number of species analysed for each taxonomic group, respectively, are shown within brackets. The identified gene duplicates are marked with letters H, S, C, T and R. Taxonomic groups are represented in different colours and labelled next to the cladogram. The known vertebrate Whole Genome Duplication (WGD) events are represented by orange circles, and the Teleost, Salmonidae and Cyprinidae specific WGDs are represented by blue circles, on top of the branches. The dashed branches represent taxonomic groups with uncertain position in the cladogram. The cladogram topology is depicted as in the Tree of Life web project [87]

*Regucalcin* can be found in non-bilaterian Porifera and Anthozoa lineages, some of the most basal lineages of the animal kingdom (Fig. 2 and Additional file 1: Fig. S1). Within the Protostomian insect species, *Regucalcin* appears to have been ancestrally duplicated within the Hemipteroid Assemblage, however the relationship between the identified *H1*, *H2* and *H3* genes could not be resolved (Fig. 2 and Additional file 1: Fig. S2). Gene *H3* is most closely related to the *D. melanogaster Regucalcin* gene chosen as a representative sequence of the remaining insects. The gene *H3* lineage depicts a high prevalence of duplications spread across many insect taxonomic groups, and some duplicates are associated with considerable gene loss events (Additional file 1: Figs. S2 to S7). Species from the dipteran Sophophora subgenus have *Regucalcin* and a known paralog of this gene, *Dca* [31, 32]. The phylogenetic approach here implemented replicates these findings and also depicts the expected *Dca* duplicates in *Drosophila ananassae* [31]. Unexpectedly, the results also show additional and yet undescribed *Dca* duplications in *Drosophila bipectinata*. On a particular note, the presence of two *Regucalcin*-like copies in *B. mori* (XP_012549788.1 and XP_004930722.1) fits the hypothesis of a novel VC synthesis pathway, identical to that of vertebrates, with the exception of the enzyme involved in the last step of the pathway [20]. Nevertheless, given the multiple functions associated with *Regucalcin*, many of them unrelated to synthesis of VC (see Background), one must be cautious regarding this interpretation.

The frequent observation of *Regucalcin* gene duplications and losses in Insecta (Additional file 1: Fig. S7) raises the issue of whether *Regucalcin* homologs are mostly non-essential genes, involved in local species adaptation. This could be the case for *D. melanogaster Dca*, since this gene has been involved in the response to cold exposure and wing size variation [25, 31–33]. Moreover, the *D. melanogaster Dca* gene has been reported as a non-essential gene [25]. Nevertheless, the *daughterless*-GAL4 (*da*-GAL4) driver used in that study to supress the expression of *D. melanogaster Dca* [25] might not have a high constitutive expression [45]. Therefore, in order to elucidate whether in insects *Regucalcin* homologs are in general non-essential genes, *Regucalcin* and *Dca* RNAi knockouts were performed using an *Actin 5 C*-GAL4 driver with ubiquitous and high constitutive expression [46]. The viability of the progeny that expresses either *Regucalcin* or *Dca* RNAi was greatly diminished (Additional file 1: Table S1), with at least 71% of the individuals not transitioning from the pupal stage. This reduced viability was particularly accentuated in the male progeny when compared to the female. Therefore, under our experimental conditions, both *Regucalcin* and *Dca* seem to be essential genes. The discrepancy between our results for *Dca* and those reported in [25], is likely explained by the distinct driver or/and the different *Dca* RNAi strains used. Given these observations, the dynamic evolution of *Regucalcin* homologs in insects is surprising.

Contrary to the recurrent gene duplications observed within insects, the *Regucalcin* gene does not seem to have been duplicated ancestrally in the remaining Non-Lophotrochozoa although it can be seen specifically duplicated in Collembola, Araneae, Platyhelminthes and Acari species (Fig. 2 and Additional file 1: Fig. S8). The presence of *Regucalcin* in the Platyhelminthes is restrained to one out of nine species analysed (*Macrostomum lignano*). This evidence suggests an event of gene loss soon after the appearance of this taxonomic group. Other *Regucalcin* loss events likely affected the Crustacea and Nematoda. Since nematodes are able to synthesize VC [18], this observation implies that VC synthesis in *C. elegans* does not occur through GULO replacement in the known animal VC biosynthetic pathway, but rather by a novel pathway that does not involve either *Regucalcin* and *GULO*. The loss of *Regucalcin* in Nematoda, further suggests that this gene does not perform other essential functions unrelated to VC synthesis, or alternatively, that this gene might have been functionally replaced by others in this group of species. Although the results suggest up to four ancestral gene duplications within the Lophotrochozoa groups (Fig. 2 and Additional file 1: Fig. S9), and subsequent multiple independent gene losses within groups, such a complex scenario is supported by a single species representative of each group. Therefore, at present, we propose a scenario similar to that for Non-Lophotrochozoa species, in which the variation in the number of gene copies is due to group specific gene duplications. These show a particularly high prevalence within Bivalvia (Additional file 1: Fig. S9).

Within deuterostomians, the *Regucalcin* evolutionary history appears to be less complex than the general scenario observed for the non-bilaterian and protostomian organisms. *Regucalcin* is present in all of the major basal deuterostomian groups, with specific duplications in Echinodermata, Hemichordata and Cephalochordata species (Fig. 2 and Additional file 1: Fig. S10). As observed for protostomians, the overall tendency for duplications observed within basal deuterostomians raises once more the issue of whether homologous genes perform similar functions or not. A two-round whole genome duplication (2R-WGD) event has likely occurred within the Craniata subphylum, after the separation of vertebrates from invertebrate chordates [47–49]. One version of this hypothesis considers that the first round of WGD affected the common ancestor of all vertebrates, while the second affected the common ancestor of jawed vertebrates, after the separation from jawless vertebrates [48, 50]. Additionally, WGD were also extrapolated at the base of teleost fish, and after within the salmonids and some cyprinids [51]. This also seems to be the case in the allotetraploid *Xenopus laevis* [52]. *Regucalcin* is present in all the vertebrate taxonomic groups analysed (Fig. 2 and Additional file 1: Fig. S11). Nevertheless, the results suggest that three out of the four putative copies that resulted from the 2R-WGD events were lost, as were some of the duplicates expected from the proposed teleost-specific WGDs. The *Regucalcin* duplicates observed in the Reptilia and Testudines, Aves and Mammalia groups (genes *R1* and *R2*) could be the result of the 2R-WGD events. Nevertheless, since these duplicates are not present in the remaining vertebrate taxonomic groups, the inference of a duplication event in the common ancestor of the Reptilia and Testudines, Aves and Mammalia groups is the most parsimonious explanation for this scenario. Interestingly, only a small fraction of mammalian species still has gene *R1*, suggesting that this duplicate likely did not acquire relevant functions and was therefore lost independently in many lineages.

### Regucalcin and Dca are involved in calcium homeostasis and oxidative stress response

As already stated, *Regucalcin* has been implicated in calcium homeostasis and oxidative stress, among others, in deuterostomians, but not in protostomians. Since the conservation of orthologous interactors is usually indicative of similar protein functional properties in distinct species [53], the comparison of the *D. melanogaster*, *M. musculus* and *H. sapiens* Regucalcin interactomes could provide clues regarding putative functions of this protein in the former species. Unexpectedly, the number of inferred *D. melanogaster* interactors (95) is substantially higher than the one obtained for *M. musculus* (three) and *H. sapiens* (nine). A significant fraction of the *D. melanogaster*, *M. musculus* and *H. sapiens* interactomes remains undiscovered [54–56], and thus this could explain the observed discrepancy. Nevertheless, it is also possible that Regucalcin may have more roles/interactors in *D. melanogaster* in comparison to the other species. Despite the small number of Regucalcin interactors reported for *M. musculus* and *H. sapiens*, one out of three *M. musculus* interactors (gene ID 22627) and four out of nine *H. sapiens* interactors (gene IDs 3094, 3336, 6647 and 9948) are also interactors in *D. melanogaster* (Additional file 1: Table S2). The shared *M. musculus* interactor (the 14-3-3 protein epsilon; gene ID 22627) is a calcium channel regulator (https://www.uniprot.org/uniprot/P62259). Moreover, one of the shared *H. sapiens* interactors (Histidine triad nucleotide-binding protein; gene ID 3094), is involved in the positive regulation of calcium-mediated signaling (https://www.uniprot.org/uniprot/P49773). These observations suggest a role of the *D. melanogaster* Regucalcin in calcium homeostasis, in contrast to previous claims of no calcium related functions [31]. Another shared *H. sapiens* interactor (Superoxide dismutase 1; gene ID 6647), which has been correlated with oxidative stress response/signaling [57] and is known to have increased activity in the presence of Regucalcin [58, 59] further suggests that the *D. melanogaster* Regucalcin may also be an apoptosis regulator [60]. The dysregulation of calcium homeostasis/signaling and apoptosis is thus likely the cause of the pupae lethality observed in *Regucalcin* RNAi experiments (see above). However, no conclusions can be drawn regarding VC synthesis with this data, since no interactor with relevant function in the known pathway was identified in either *M. musculus* or *H. sapiens.* For Dca there is no protein-protein interaction data. Nevertheless, the high similarity between Regucalcin and Dca suggests that they might share interactors. The protein docking inferences of Regucalcin and Dca versus the 14-3-3 protein epsilon, Histidine triad nucleotide-binding protein 1 and Superoxide dismutase 1 here made show that this is a possibility, although the predicted Dca and Regucalcin interacting regions are different (Additional file 1: Fig. S12). Indeed, in two out of three cases, Dca has more interacting amino acids than Regucalcin (39, 52 and 37 for Dca versus 41, 45 and 34 for Regucalcin, respectively), although the interacting surface of *D. melanogaster* Regucalcin is the same as that observed in humans for the same three interactors (Additional file 1: Fig. S13). A putative role of *Dca* in calcium homeostasis could explain the involvement of this gene in wing size variation, since organ development depends on coordinated cell-cell communication that in turn requires signal integration among multiple pathways, relying on second messengers such as calcium ions [61].

### Inferences on PSS

The location of positively selected sites (PSS) on predicted protein structures can give insight into which protein regions are most often the target of positively selected amino acid changes. While the presence of PSS around the lid/active site of Regucalcin homologs suggests a possible effect on the modulation of its gluconolactonase activity, clusters of PSS located elsewhere suggest a possible effect on the modulation of the interaction strength between Regucalcin and its interactors.

**Fig. 3:**
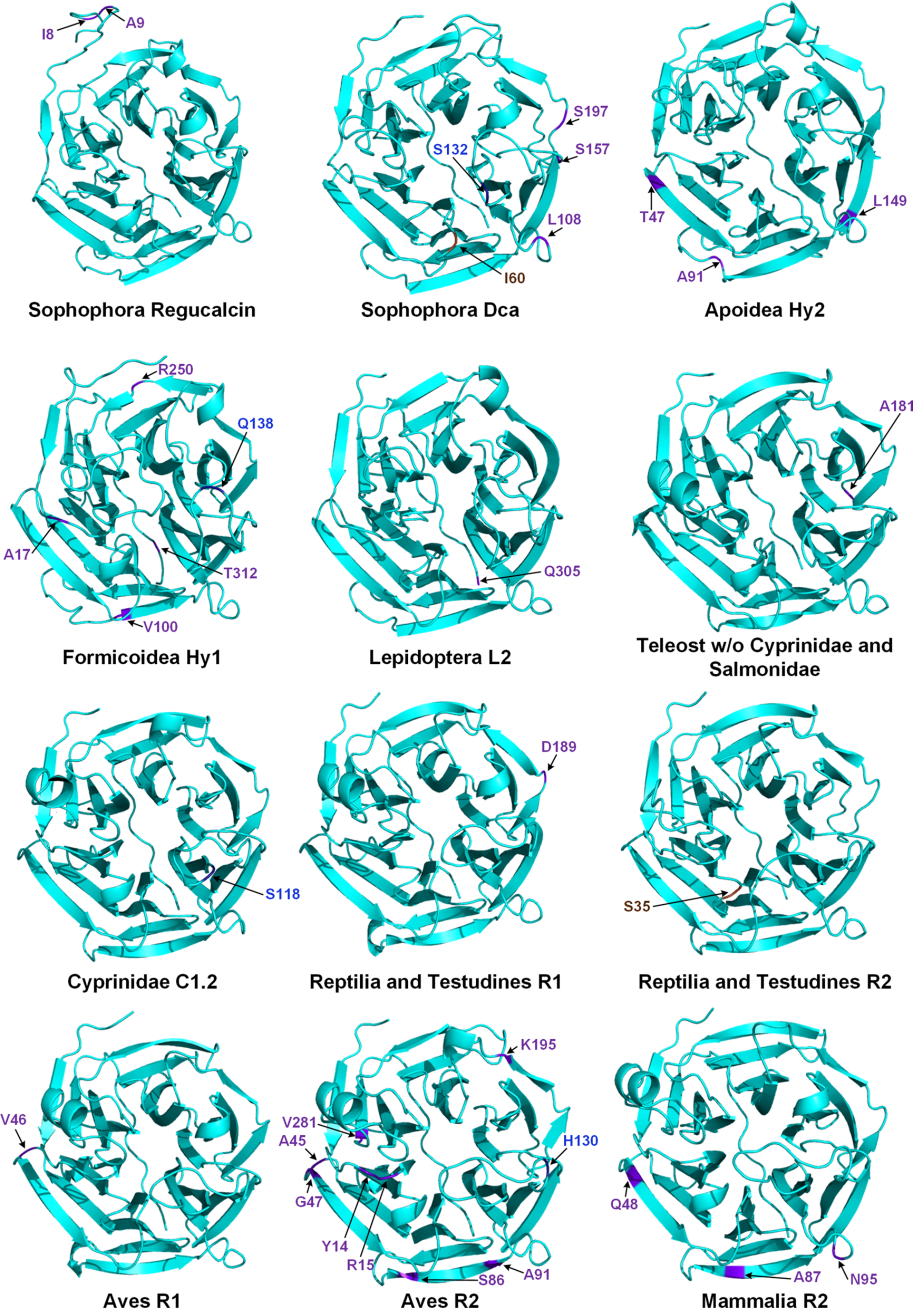
Distribution of PSS on predicted I-TASSER protein structures. The representative protein sequences used for the inferences were *D. melanogaster* AAN09306.2, *D. melanogaster* AGB95961.1, *Bombus terrestris* XP_020722941.1, *Acromyrmex echinatior* XP_011068254.1, *Bombyx mori* XP_012549788.1, *Poecilia mexicana* XP_014859494.1, *Danio rerio* NP_991309.1, *Gekko japonicus* XP_015266456.1, *G. japonicus* XP_015266450.1, *Coturnix japonica* XP_015706795.1, *C. japonica* XP_015706720.1 and *H*. sapiens NP_690608.1, respectively from the top left to the bottom right. The overall predicted structures are highlighted in cyan, while the identified PSS are marked in dark blue, brown and purple according to their proximity to the active site (close to distant, respectively) and tagged with the corresponding amino acid symbol and position in the reference sequence

In total, 33 PSS are here inferred at the protein surface of Regucalcin homologs, in 12 animal lineages (Fig. 3), one of which being the Sophophora Dca lineage where evidence for positive selection has been previously uncovered [31]. Four PSS are found in the lid region of the inferred protein models (Sophophora Dca S^132^, Formicoidea Hy1 Q^138^, Cyprinidae C1.2 S^118^ and Aves R2 H^130^, labeled in dark blue in Fig. 3) and two surrounding the active site (Sophophora Dca I^60^ and Reptilia and Testudines R2 S^35^, labeled in brown in Fig. 3). These observations suggest that the modulation of Regucalcin´s gluconolactonase activity might be important for a wide variety of species, and that the gluconolactonase activity is important for *D. melanogaster*´s Dca function.

**Fig. 4:**
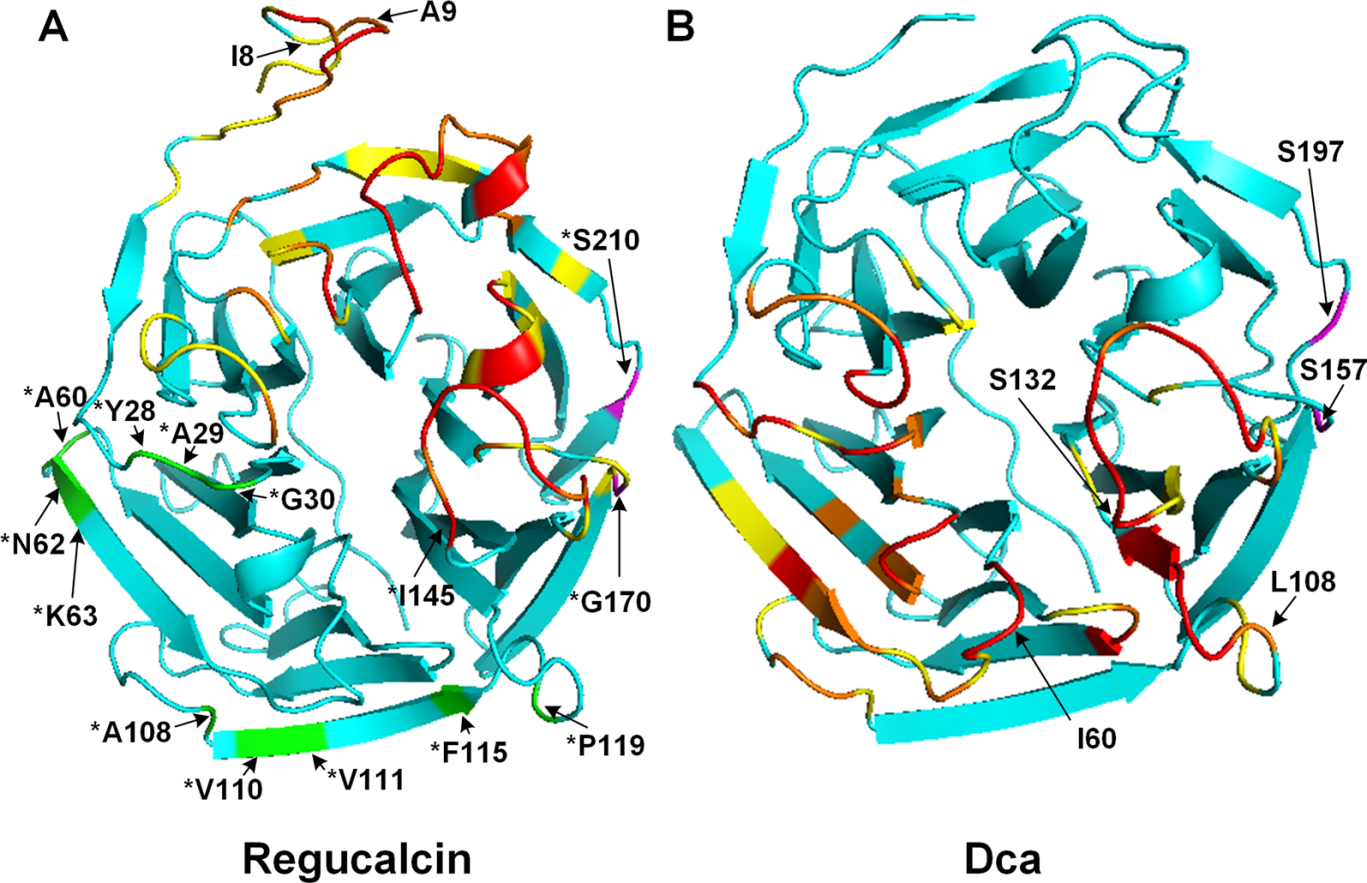
Distribution of predicted interacting residues on the *D. melanogaster* Regucalcin AAN09306.2 (**A**) and Dca AGB95961.1 (**B**) models. The overall predicted structures are highlighted in cyan, while the interacting residues are marked in yellow (one predicted interaction), orange (two predicted interactions) and red (three predicted interactions). The previously inferred PSS positions are represented by the corresponding amino acid symbol and sequence position in both proteins. The I^145^,G^170^ and S^210^ Regucalcin positions (homologous to the Dca S^132^,S^157^ and S^197^) are also displayed, as well as the homolog amino acids relative to the remaining datasets PSSs, with a “*” prefix. The Dca PSSs that were not correlated with the interaction regions (S^157^ and S^197^), as well as their homologues in Regucalcin (G^170^ and S^210^), are highlighted in magenta, while the remaining datasets PSSs homologs are shown in green. Although the distribution of interacting surfaces is distinct, the lid and active site regions of Regucalcin and Dca appear to be conserved

Twenty-seven PSS can be seen spread across the remaining regions of the protein models (labeled in purple in Fig. 3; Additional file 1: Table S3). Since they are away from the lid region and the active site, it seems likely that they define interaction surfaces with Regucalcin interactors. This is a strong possibility since in *D. melanogaster* Regucalcin has, so far, been reported to interact with 95 other proteins (see EvoPPI 1.0; *D*. *melanogaster* same species query; [62]). Nevertheless, only two *D. melanogaster* Regucalcin PSS (I^8^ and A^9^) are contained in the Regucalcin interacting region here inferred (Fig. 4 A and Additional file 1: Fig. S12). Surprisingly, the other PSS are clustered around the predicted interacting surface for Dca (Fig. 4 A and Additional file 1: Fig. S12). As for *D. melanogaster* Dca, two out of the three PSS not found in the lid region of the inferred protein models or surrounding the active site, are found in the interface region here predicted for Regucalcin (S^157^ and S^197^; Fig. 4B). It is thus possible that there is more than one interface region in both Regucalcin and Dca. PSS away from the lid and the active site may be involved in the destabilization/strengthening of ancestral PPIs, or even be involved in the establishment of novel PPIs after the duplication event.

### *SVCT* evolution in animals

The observation that *B. mori* is unable to synthesize enough VC during larval stages 3-LE up to 5-LE for its needs [20], implies the presence of a functional VC transporter in the gut tissues for the dietary uptake of this nutrient. In vertebrate species, this is the primary role of the SVCT1 protein [36]. A VC transporter with a function similar to SVCT1 protein should thus be present in *B. mori*, as well as in the many other protostomian species that are believed to be unable to synthesize VC. It should, however, be noted that in vertebrates not all SVCT are able to transport VC [36]. Therefore, the presence of a *SVCT-*like gene by itself cannot be taken as evidence for VC transport. The phylogenetic inferences here performed are also an opportunity to elucidate the origin and evolution of *SVCT* genes.

**Fig. 5:**
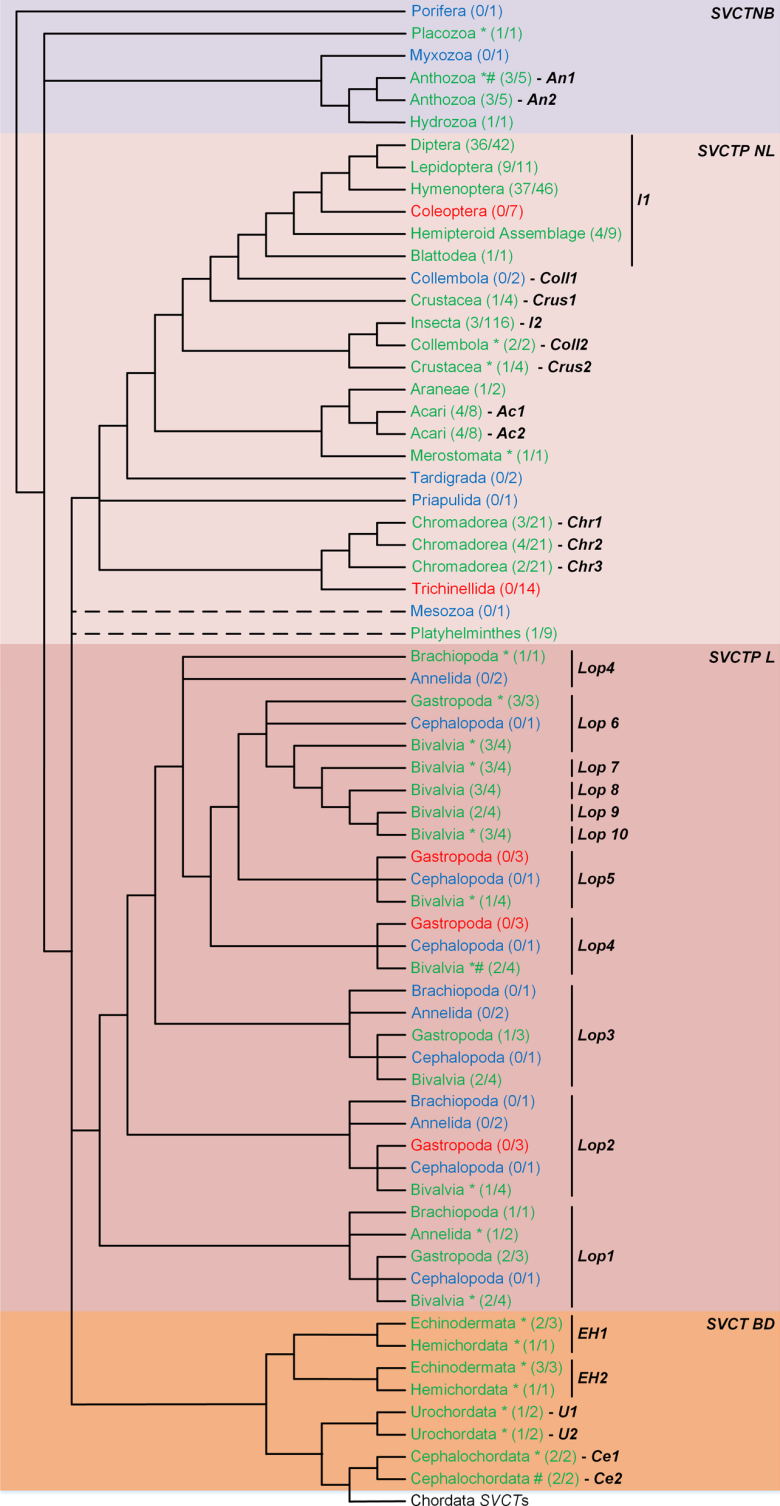
Proposed *SVCT* gene evolution in Non-Bilateria (*SVCTNB*), protostomian Non-Lophotrochozoa (*SVCTP NL*), protostomian Lophotrochozoa (*SVCTP L*) and basal deuterostomian (*SVCT BD*) species. Gene presence is highlighted in green, absence in red and uncertainty regarding gene loss/presence in light blue. Gene duplications affecting a single species of a genus are marked with a *, while those which affected two or more species from a genus within a defined taxonomic group are marked with a #. The number of species present in the final dataset and total number of species analysed for each taxonomic group can be seen within parentheses, respectively. The inferred gene duplicates are represented next to the taxonomic group names. The cladogram topology is depicted as in the Tree of Life web project [87]

The more basal taxonomic groups depict an overall complex evolutionary history. The non-bilaterian *SVCT* (*SVCTNB*) gene appears independently duplicated in the represented taxonomic groups, with the exception of the Hydrozoa phylum. (Fig. 5 and Additional file 1: Fig. S14).

The Non-Lophotrochozoa *SVCT-protostomian* gene (*SVCTP NL*) might have been duplicated in the ancestral of the Pancrustacea, since two genes can be inferred for the Insecta and Crustacea groups (Fig. 5 and Additional file 1: Fig. S15). Nevertheless, the vast majority of insect species only have one copy (Gene *I1*). Therefore, it is likely that the single *SVCTP NL* gene found in *B. mori* is the VC transporter in this species. Curiously, the Coleoptera species seem to have lost the *SVCTP NL* gene. In the Nematoda taxonomic group, the *SVCTP NL* gene is also missing in 29 out of the 35 species analysed (Fig. 5 and Additional file 1: Fig. S15), including *C. elegans*, where VC synthesis has been described [18], and all the Trichinellida species. The loss of a SVCTP transporter in the coleopterans and many nematodes suggests that such species do not rely on an external VC source, implying that they must be able to synthesize their VC. Nevertheless, the possibility that a different VC transporter is used cannot be completely ruled out. Moreover, it suggests that in these lineages SVCTP has a dedicated function, such as VC transport, rather than a generalized function, since the loss of a gene performing multiple functions is likely lethal. The Platyhleminthes also suffered a notable *SVCTP NL* loss, since this gene is only present in one out of nine species analysed (Fig. 5 and Additional file 1: Fig. S15).

The *SVCTP* evolutionary history in the Lophotrochozoa (*SVCT L*) has a remarkably difficult interpretation, mainly due to the numerous Bivalvia duplicates present (Fig. 5 and Additional file 1: Fig. S16). Indeed, the distribution pattern of the Bivalvia sequences implies up to 10 distinct genes (*Lop1* to *Lop10*). It is unclear whether each of the multiple genes found in Lophotrochozoa is dedicated to a specialized function as observed in vertebrates [36].

The results regarding the basal deuterostomians *SVCT* (*SVCT BD*) should be interpreted with caution, since the phylogeny obtained depicts considerable branch politomy (Additional file 1: Fig. S17). Nevertheless, one possible hypothesis implies the occurrence of a *SVCT BD* duplication in the common ancestor of Echinodermata and Hemichordata, and independent duplications in the ancestors of the Urochordata and Cephalochordata, respectively (Fig. 5 and Additional file 1: Fig. S17). If so, the duplicates themselves have been further duplicated in specific lineages/species.

**Fig. 6:**
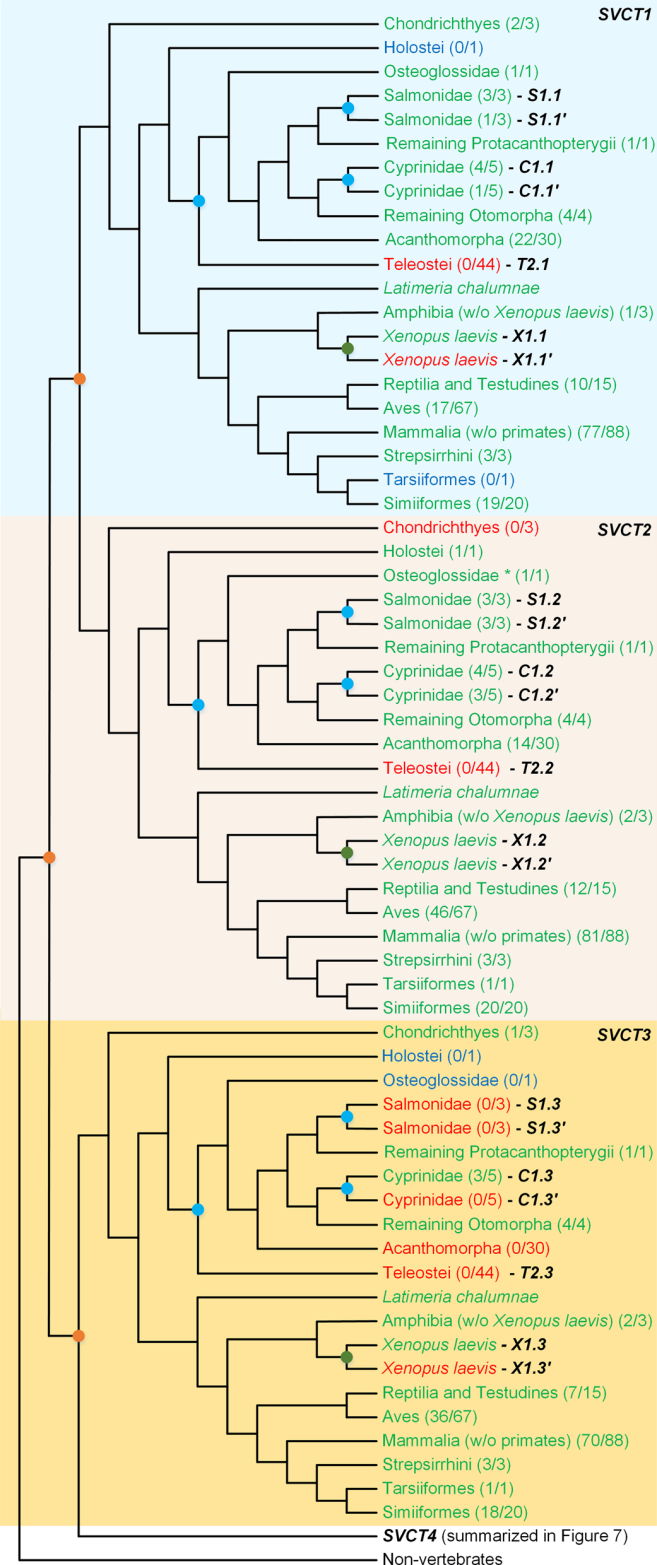
Proposed *SVCT1*, *SVCT2* and *SVCT3* gene evolution in vertebrate species. Gene presence is marked in green, absence in red and uncertainty regarding gene loss/presence in blue. The number of species present in the final dataset and total number of species analysed for each taxonomic group can be seen within parentheses, respectively. The identified gene duplicates are represented next to the cladogram. The known vertebrate WGD events are represented by orange circles in the branches, the Teleost, Salmonidae and Cyprinidae specific WGDs are represented by blue circles and the *X. laevis* WGD by green circles. The cladogram topology is depicted as in the Tree of Life web project [87]

Concerning the vertebrate species, the *SVCT1* gene is present in all the main lineages analysed (Fig. 6 and Additional file 1: Fig. S18). This gene is duplicated in salmonids and cyprinids, likely due to the two independent WGD events known to have affected the salmonid and some cyprinid species [51]. Although this gene is present in all the main vertebrate groups, it is only present in 17 out of 67 species within the Aves class, suggesting a considerable number of loss events in this lineage. Since SVCT1 is involved in VC uptake, this observation suggests that many Aves species do not rely on an external VC source. This is surprising, since several birds are known to have lost *GULO* [13, 21], and are theoretically incapable of synthesizing VC through the known animal pathway. From our data, 13 out of 67 species spread across nine Aves orders exhibit a lack of both *GULO* and *SVCT1*. It is, however, possible that in these 13 species *GULO* may be badly annotated or present in unplaced or non-sequenced regions of the genome.

The *SVCT2* gene appears to be present in all the main vertebrate lineages, apart from the Monotremata, Xenarthra and Chondrichthyes (Fig. 6 and Additional file 1: Fig. S19). Evidence for *SVCT2* gene duplications can be observed within the Actinopteri, possibly explained by the WGD events that occurred in the Salmonidae and Cyprinidae lineages [51], and an independent specific gene duplication in the Osteoglossidae family. Regarding the Amphibia, the extrapolated WGD that affected *X. laevis* [52] can explain the single duplication observed. Furthermore, the *SVCT2* gene presence within the Aves class is remarkably more accentuated when compared to that observed for *SVCT1*, as it is detected in 47 out of 67 species analysed.

The *SVCT3* gene does not seem to be closely related with either *SVCT1*, *SVCT2* and *SVCT4*. The somewhat distant phylogenetic relation with *SVCT1* and *SVCT2* was not surprising, as it was previously hypothesized that *SVCT3* diverged from these genes at an early evolutionary stage [36]. Nevertheless, the distant phylogenetic relation of this gene relative to *SVCT4* was not obvious, given that the SVCT3 transporter is proposed as the functional replacement of SVCT4 in species where *SVCT4* is pseudogenized [36]. However, a previous report revealed that the *SVCT3* sequences have unstable phylogenetic positions, even when using several prediction models [41]. An accelerated rate of nucleotide substitutions could be due, for instance, to a period of neutral evolution, before neo-functionalization. Although a conclusive explanation for this phenomenon cannot be extrapolated from this data, this hypothesis is compatible with the idea that *SVCT3* and *SVCT4* may derive from a single ancestral lineage affected by an event of WGD. Curiously, the *SVCT3* gene does not seem to be duplicated in any taxonomic group (Fig. 6 and Additional file 1: Fig. S20). In addition, the majority of Actinopteri (37 of 45) and almost half of the Aves species (31 of 67) seem to have lost the *SVCT3* gene. Although the loss of *SVCT3* in a large number of teleost fish was previously identified [41], to our knowledge it was not yet reported concerning the Aves class. In mammals, SVCT3 is known to be mainly present in the renal proximal straight tubule segments of the kidney, and is likely involved in molecule reabsorption processes [36], and thus *SVCT3* gene losses may be correlated with differences in the excretion mechanism of the species being compared.

**Fig. 7:**
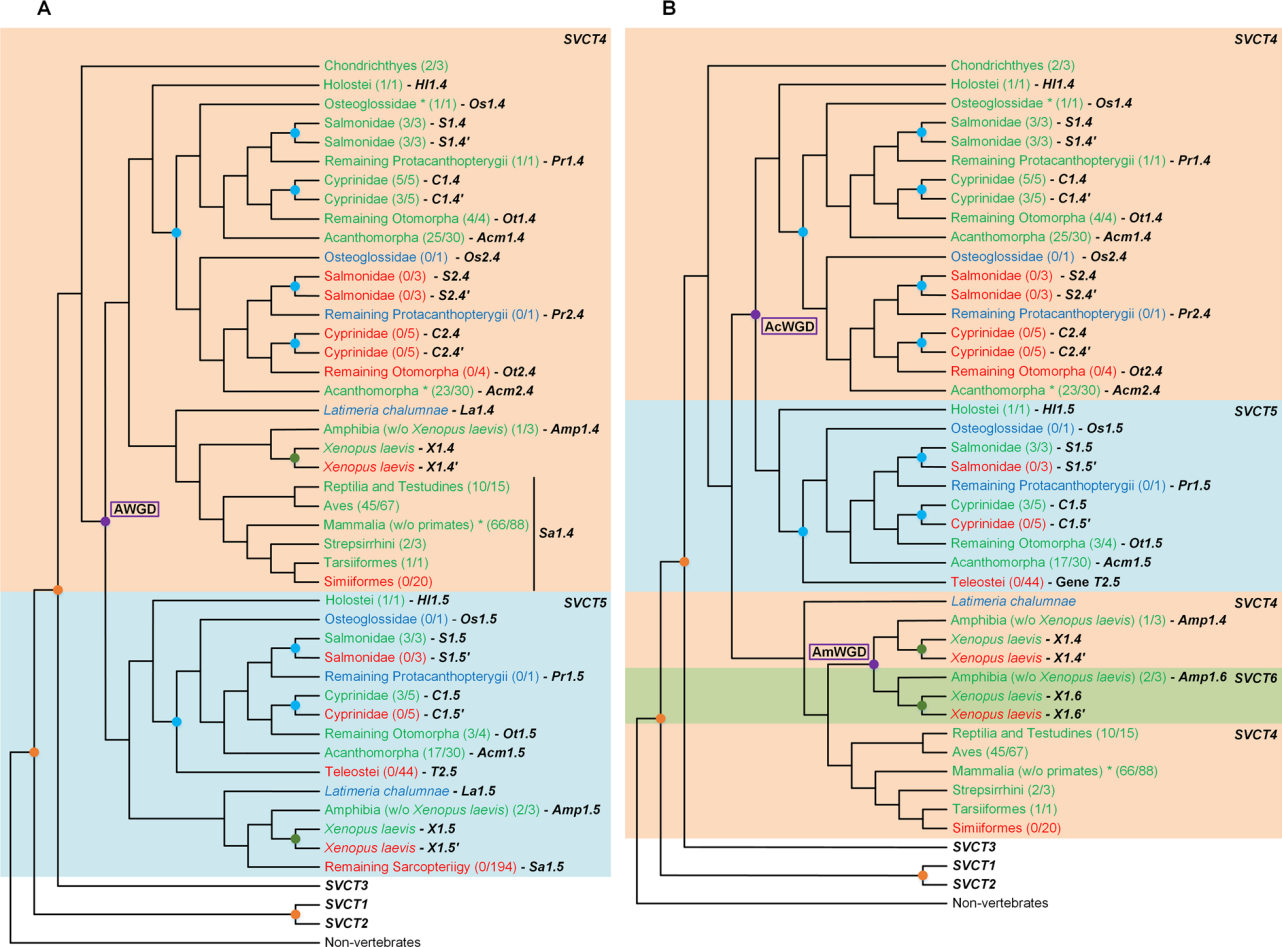
Proposed *SVCT4* gene evolution in vertebrate species. Hypothesis (**A**) considers a possible ancestral duplication event (AWGD) that preceded all vertebrates after the separation from the Chondrichthyes, originating a putative *SVCT5.* Hypothesis (**B**) considers that two independent duplication events might have affected the Actinopteri and Amphibia groups (AcWGD and AmWGD, respectively), originating the *SVCT5* and *SVCT6* genes. *SVCT4, SVCT5* and *SVCT6* presence is highlighted in green, absence in red and uncertainty regarding gene loss/presence in light blue. The number of species present in the final dataset and total number of species analysed for each taxonomic group can be seen within parentheses, respectively. The identified gene duplicates are represented next to the cladogram. The known vertebrate WGD events are represented by orange circles in the branches, the Teleost, Salmonidae and Cyprinidae specific WGDs are represented by blue circles and the *X. laevis* WGD by green circles. The hypothesized duplication events are highlighted in purple. The cladogram topology is depicted as in the Tree of Life web project [87]

*SVCT4* is present within most of the vertebrate taxonomic groups (Fig. 7 A and B, and Additional file 1: Fig. S21). This gene is duplicated in 23 Acanthomorpha species, likely because of the teleost-specific WGD that is proposed to have taken place in the common ancestor of all teleosts [51, 63]. This hypothesis is compatible with the absence of duplications in *L. oculatus*, which suggests that the duplication event did not affect species from the Teleost sister group, Holostei. In addition, the *SVCT4* gene present in several Salmonidae and Cyprinidae species seem to have been affected by lineage-specific WGD events. A previous report stated that as in humans, several higher primate species could have lost the *SVCT4* gene [44], a finding that is here replicated. Nevertheless, the *SVCT4* gene is still found in three primate species, namely *Carlito syrichta* (Haplorrhini), *Propithecus coquereli* and *Microcebus murinus* (both Strepsirrhini). It is important to note that although *C. syrichta* belongs to the Haplorrhini suborder (where *SVCT4* is mostly loss or pseudogenized), it does not belong to the Simiiformes group (higher primates), but to the Tarsiiformes infraorder [64]. Given this evidence, it is likely that the *SVCT4* gene may have been lost in the Simiiformes, after the split of the Tarsiiformes. The initial *SVCT4* phylogenetic inference revealed an isolated cluster of 27 Actinopteri and three Amphibia coding sequences relative to the identified *SVCT4* sequences, leading to the creation of the *SVCT5* phylogeny (Figure S22). This phylogeny points to two possible scenarios: (i) a putative *SVCT4* duplication event that preceded the split of the Actinopteri and Amphibia taxonomic groups that originates the *SVCT5* gene (Fig. 7A), or (ii) two specific and independent *SVCT4* duplication events at the base of the Actinopteri and Amphibia taxonomic groups that originated two distinct genes similar to *SVCT4* (arbitrarily designated as *SVCT5* and *SVCT6*, respectively; Fig. 7B). Regardless of the evolutionary origin, these sequences appear to be clearly distinguishable from the known vertebrate *SVCT* genes and may represent novel nucleobase-ascorbate transporters yet uncharacterized.

### SVCT functions in non-deuterostomian species

The inferences made on the evolutionary history of animal SVCTs (see above), in combination with the functional *B. mori* studies [20], suggests that SVCTP is able to perform VC transport. Therefore, as performed for Regucalcin, the comparison of the *D. melanogaster* SVCTP interactome with that of vertebrate SVCTs could provide evidence to support this hypothesis. It should be noted that although 25, 11, and 2 interactors have been reported for *H. sapiens* SVCT1, SVCT2 and SVCT3, there is no overlap between these and the *D. melanogaster* SVCTP paralog interactors (Additional file 1: Table S4). Moreover, no SVCT interactors have been reported for *M. musculus*. Therefore, at present, a given function cannot be correlated with the presence of a given interactor.

Amino acid motifs may also give information on substrate specificity, such as the SSSP motif reported in the literature [41]. This amino acid motif is only found in SVCT1 and SVCT2 proteins, the two SVCTs so far reported to be able to transport VC. None of the basal deuterostomian, protostomian and non-bilaterian SVCT sequences have this motif. It should be noted that in the SSSP motif, the most important amino acids for substrate affinity (the second serine and the proline; [41]) are not simultaneously found in any sequence from non-deuterostomian and basal deuterostomian species. Non-bilaterian species from the Placozoa, Hydrozoa and Anthozoa phyla, as well as the majority of the basal deuterostomian species, have, however, one amino acid motif that is present in the SVCT4 transporter (SYSE), suggesting that these SVCTs are able to transport nucleobases. Insect species present the consensus motif TFGE, which is remarkably similar to the *E. coli* Uracil permease motif (TYGE; [41]). In *Drosophila*, uracil secreted by gut pathogens is crucial to activate the intestinal dual oxidase immune response at the midgut level [65]. This implies that a transporter of this nucleobase at the gut level might play an important role in host/pathogen interaction, in addition to the inferred uracil homeostasis functions. Although the *D. melanogaster SVCTP* has low expression in the midgut, it has moderate expression in the hindgut and high expression on the Malphigian tubules (https://flybase.org/reports/FBgn0037807), and therefore might intervene in such process in these specific tissues. The models presented in [65] consider only the recognition of uracil by surface receptors in enterocytes, but the transport of uracil and subsequent intracellular recognition of this nucleobase is also a possibility, since immune responses can also be mediated by cytoplasmic receptors [66]. Although the motif analyses suggest that SVCTNB and SVCTP are able to perform the transport of nucleobases only, they must also be able to transport VC given that *B. mori* is unable to synthesize enough VC during larval stages 3-LE up to 5-LE for its needs, and no other VC transporter is known [20].

## Discussion

### ***Regucalcin***, ***GULO*and*SVCT***: **a history of neofunctionalization, pseudogenization, and subfunctionalization**

**Fig. 8:**
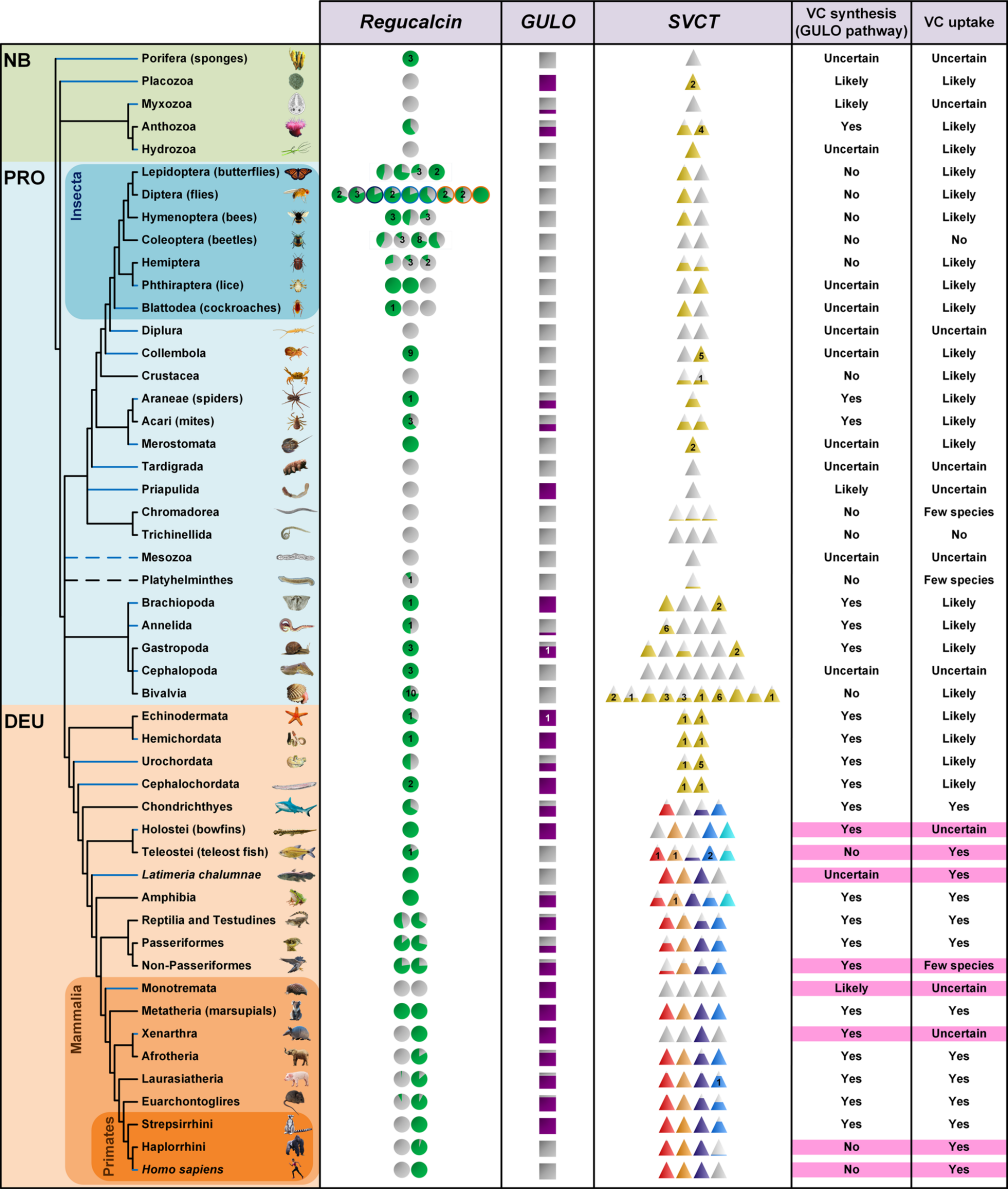
Patterns of *Regucalcin*, *GULO* and *SVCT* gene presence/absence within the animal kingdom. NB, PRO and DEU represent the non-bilaterian, protostomian and deuterostomian species, respectively. Circles are representative of the *Regucalcin* gene, while squares and triangles represent the *GULO* and *SVCT* genes, respectively. The figures’ fill is indicative of the percentage of species in which the given gene was identified relative to the total number of analysed species, while grey figures represent complete gene absence. The estimated GULO presence/absence frequency is based on previous reports [19, 21] where species for which no gene annotation is available, and thus where a manual gene annotation was performed, were also considered. The number of figures for each gene indicates the identified ancestral duplicates within each group/species. Numbers highlighted in the figures represent the inferred specific gene duplications found within the lineages. The purple colour outline within the *Regucalcin* Diptera section of the image represents the Sophophora *Dca* gene, while the dark blue, blue and orange represent the Drosophila *Regucalcin*, the Tephritidae *Regucalcin-like* and Culicidae *Regucalcin-like* genes, respectively. The four known *SVCT* genes within the vertebrates lineage are represented in red (*SVCT*1), orange (*SVCT*2), dark blue (*SVCT*3) and blue (*SVCT*4), while the putative *SVCT*5 is highlighted in light blue. The *SVCTNB*, *SVCTP NL*, *SVCTP L* and *SVCT BD* genes are represented in burnt yellow. The blue branches in the cladogram highlight the taxonomic groups represented by less than three species in the analyses. The last two columns summarize the current hypothesis regarding VC synthesis (via GULO pathway) and uptake from diet, respectively. Within these, the information highlighted in light pink represents possible correlations between loss of *SVCT1* and *GULO* conservation, and vice-versa. The cladogram topology is depicted as in the Tree of Life web project [87]. The Euarchontoglires and Haplorrhini groups in the cladogram do not consider primates and *H. sapiens*, respectively, due to the subsequent taxa expansions

A summary of the *Regucalcin*, *GULO* (data from [19, 21]) and *SVCT* presence/absence along the animal tree is shown in Fig. 8. For all lineages where there is enough data to be confident that a given gene is missing, when *GULO* is present, there is at least one *Regucalcin* homolog also present at a similar or higher frequency, with the exception of the Gastropoda. Therefore, species where a *GULO* gene is found and that belong to these lineages are inferred to synthesize VC through the described animal pathway. Gastropoda may also use the described animal pathway to synthesize VC, although in this case we have to assume that different Gastropoda species use different *Regucalcin*-like genes that belong to different gene lineages. In Monotremata, Priapulida, Myxozoa and Placozoa, a *GULO* gene has been identified but not a *Regucalcin* gene. Nevertheless, given that a single species is being analysed in each case, and given the possibility that genomes may not have been fully sequenced, at this point, we still consider that it is likely that these species synthesize VC through the described animal pathway.

As suggested before, *GULO* is likely only involved in the last step of the VC synthesis [13]. Despite the 2R-WGD event that likely occurred within the Craniata subphylum, after the separation of vertebrates from invertebrate chordates [47–49], the WGD that is proposed to have taken place in the common ancestor of all teleosts [51, 63], the two independent WGD that affected Salmonidae and Cyprinidae [51], and the WGD that affected *X. laevis* [52], *GULO* is almost always found as a single copy gene. As such, it seems that there is no advantage in having high L-gulonolactone oxidase activity levels. *GULO* is prone to pseudogenization in the lineages where VC is acquired through a VC-rich diet, which is likely the case of Haplorrhini primates, Passeriformes, Teleostei fish and some bats (included in the Laurasiatheria group). This could also be the case of *L. chalumnae*, although in this case we cannot be certain that *GULO* was lost in this lineage since a single species was analysed. Nevertheless, if true, given that Teleostei species are more closely related to Holostei species than to *L. chalumnae*, this would imply another independent loss of the ability to synthesize VC. This is a possibility since multiple independent *GULO* losses are common even at short evolutionary scales, as observed in bats [67].

Species from the Haplorrhini and Teleostei groups are known to develop scurvy in the absence of a VC rich diet [9, 68]. *SVCT1* is the only vertebrate gene that has been implicated in specific VC uptake from the diet [36, 42], and thus is an essential gene in species that lost the ability to synthesize VC. As expected, in Haplorrhini primates, Passeriformes and Teleostei fish, a *SVCT1* gene is almost always found. *SVCT1* can only be found in a few non-Passeriformes species, which implies that many of the analysed non-Passeriformes are unable to uptake VC from the diet. As such, they must synthesize their own VC, which is in agreement with the identification of a *GULO* gene in most non-Passeriformes species (Fig. 8). The loss of the *SVCT1* gene means that *GULO* is now an essential gene in this lineage, which can no longer be lost by pseudogenization. It is known that the basally branching actinopterygian fishes can synthesize VC in their kidneys [69], as amphibians, reptiles and the basally branching birds do [13], which is in agreement with the identification of a single copy of *GULO* and *Regucalcin* in Holostei fish (Fig. 8). Although a single species is being considered, and as such it is impossible to infer with certainty any gene loss in the Holostei lineage, the absence of a *SVCT1* gene, as here observed, would explain why *GULO* cannot be lost in this lineage.

Although a *GULO* gene is present in echinoderms, sea cucumbers have a daily VC requirement in their diet similar to that found in Teleostei fish [70]. Therefore, at least one of the two *SVCT*s found in these basal deuterostomian species must be able to transport VC. This observation suggests that SVCTs were already able to transport VC before the two-round whole genome duplication (2R-WGD) event that likely occurred within the Craniata subphylum, after the separation of vertebrates from invertebrate chordates [47–49]. This is further supported by the observation that the insect *B. mori* is unable to synthesize enough VC during larval stages 3-LE up to 5-LE for its needs [20], implying that the only SVCTP present in this species must be able to transport VC. Moreover, in bees, the dietary supplementation of VC increases resistance to infection, showing that VC must have been transported [71]. Therefore, despite the inferences here made based on amino acid patterns that SVCTPs are involved in the transport of nucleobases only, they must also transport VC. As such, the 2R-WGD event likely resulted in subfunctionalization and not in neofunctionalization. In some fish and amphibian species yet undescribed *SVCT* genes are here found, which might represent further subfunctionalization or neofunctionalization processes.

In almost all protostomian and non-bilaterian groups represented by more than one species and where no *GULO* gene was found, one *SVCT* gene is always found. The clear exceptions concerns the nematodes and coleopterans, since there is no *SVCTP* in *C. elegans* and the analysed species from the Trichinellida and Coleoptera orders. Therefore, these species must exclusively rely on the synthesis of VC to meet their nutritional needs. In agreement with this observation, *C. elegans* has been shown to synthesize VC [18]. Since there is no *GULO* and *Regucalcin* in these nematode species, VC must be synthesized through a novel pathway quite different from the one so far known for animals. Coleopterans have multiple *Regucalcin-*like genes, and as such, should theoretically be capable of synthesizing VC through one of the two hypothesized insect pathways. Since the coleopterans have a homolog of the *B. mori Gulo-like* gene while the dipterans such as *D. melanogaster* do not (data not shown), it is more parsimonious to assume that the coleopterans likely rely on the *B. mori* pathway to obtain this nutrient.

Besides VC synthesis, *Regucalcin* is also likely involved in calcium homeostasis, and oxidative stress response in Deuterostomes, Protostomes and non-Bilateria animals. Whether *Regucalcin’s* gluconolactonase activity is essential for calcium homeostasis is unclear, since this role may be played through the inferred binding surface of Regucalcin-like proteins. In animals, with the clear exceptions of Insects and the Lophotrochozoa group, *Regucalcin* tends to be a single copy gene. Considering the aforementioned WGD events that likely occurred in vertebrates relative to the results here obtained, it can be concluded that the most likely fate of a *Regucalcin* duplicate is to be lost. The *Regucalcin* gene duplication inferred at the base of the reptiles/birds/mammals lineage also implies multiple independent gene losses of the *Regucalcin* duplicate. The most parsimonious explanation for these observations is that, generally speaking, despite its many roles, *Regucalcin* rarely evolves a new function. Insects are a clear exception, since *Regucalcin* homologues may have acquired a new function (neofunctionalization) related to the receptor-mediated uptake of hexamerin storage proteins from insect haemolymph by fat body cells, a feature that is only found in this group of species [72]. While in insects it is unclear whether *Regucalcin* plays any role in VC synthesis (with the possible exception of *B. mori*; [20]), this gene is likely involved in this process in the Brachiopoda, Annelida and Gastropoda, since *GULO* is present in some species from these groups, and as such, no *Regucalcin* neofunctionalization is expected. In Bivalvia, however, where no *GULO* gene has been found, it is possible that *Regucalcin* evolved new functions.

## Conclusions

In animals, VC is an essential nutrient that can be synthesized/acquired from the diet. The gene involved in the last step of the only described animal synthesis pathway (*GULO*) has been lost multiple times during evolution in non-bilateria, protostomes and deuterostomes. Species that lack *GULO* are dependent on the VC present in the diet, which implies the presence of a functional VC transporter. It is here argued that this is mainly the case, although there are clear exceptions such as *C. elegans*, where a new VC synthesis pathway must be present. Here, it is also shown why in some basal animal lineages the ability to synthesize VC cannot be lost, even in the presence of VC rich diets, due to the loss of the VC transporter. Homologs of the gene involved in the penultimate step of VC synthesis (*Regucalcin*) are shown to be likely involved in VC synthesis, calcium homeostasis and the oxidative stress response in all animals, and to have acquired new roles during evolution in some taxonomic groups such as insects. Despite its multiple functions, there is no *Regucalcin* gene in *C. elegans*. Therefore, the presence of a *Regucalcin* gene alone cannot be taken as an indication of a partially conserved VC synthesis pathway in species where there is no *GULO* but VC synthesis occurs, such as insects.

## Methods

### *Regucalcin* and *SVCT* phylogenetic analyses

An adaptation of the protocol described in [21] was used to obtain the sequence sets for the phylogenetic inferences. CDS were downloaded from the NCBI RefSeq database, and sequence operations performed using the SEquence DAtaset builder’s (SEDA) software [73]. The protein queries for the initial tblastn were *H. sapiens* Regucalcin (accession number NP_690608.1) and three distinct SVCT sequences, namely *H. sapiens* SVCT1 (NP_689898.2; XP_011542067.1), SVCT2 (CAB58120.1; NP_005107.4) and SVCT3 (NP_001138362.1). To obtain the final consensus *SVCT* dataset, the three distinct *SVCT1*, *SVCT2* and *SVCT3* output datasets were processed separately, subsequently merged, and then two additional operations, namely, “remove redundant sequences” and “remove isoforms”, performed. Two *Manacus vitelinus* CDS with the same header and accession number (XP_008924532.1) but different nucleotide sequences (99% identical) caused issues with the isoform removal step when processing the *SVCT* datasets. This problem was solved by the random removal of one of the sequences. For both *Regucalcin* and *SVCT*, the complete list of sequences that have been removed is presented in Additional file 1: Table S5. Bayesian phylogenetic inferences were performed using ADOPS [74], using 1,000,000 generations for *Regucalcin* and 5,000,000 for *SVCT*s. The first 2500 *Regucalcin* and 12,500 *SVCT* samples were discarded (burn-in).

The phylogeny obtained using all *Regucalcin* sequences, had poor branch support values, due to the use of very divergent sequences (Additional file 1: Table S5). Therefore, the dataset was also divided into smaller datasets in order to analyze sequences from Non-Bilateria, Hemipteroid Assemblage and Blattodea, Coleoptera, Hymenoptera, Diptera, Lepidoptera, Non-Lophotrochozoa (without Insects), Lophotrochozoa, Basal Deuterostomia and Vertebrates species, independently of sequences from the other groups. Moreover, sequences associated with very long branches were also removed from the datasets. The resulting datasets were then analysed as described above. Convergence was achieved in all cases. The *H. sapiens Regucalcin* sequence (NP_690608.1) was used as root outgroup for the Non-Bilateria, protostomian and basal deuterostomian datasets, while the urochordate *C. intestinalis* sequence (XP_002120764.1) was used for the deuterostomian dataset.

The *SVCT* phylogeny did not converge for all the model parameters, likely due to the inherent difficulty in the alignment of sequences of distinct genes. To overcome this technical issue, sequences that caused abnormal branch lengths in the tree were removed (Additional file 1: Table S5), and the dataset was subdivided into smaller datasets representative of all *SVCT* genes here found *(SVCT1*, *SVCT2*, *SVCT3*, *SVCT4* and *SVCT5*), as well as four subsets, representative of non-bilaterians (*SVCTNB*), protostomian Lophotrochozoa (*SVCTP L*), protostomian Non-Lophotrochozoa (*SVCTP NL*) and basal deuterostomians (*SVCT BD*). Outgroup sequences were added to each dataset, from the following sequence pool: *D. melanogaster SVCTP* (AAF54519.1), *H. sapiens SVCT1* (XP_011542067.1), *H. sapiens SVCT2* (NP_005107.4), *H. sapiens SVCT3* (NP_001138362.1), *M. musculus SVCT4* (XP_006506197.1), *Trichoplax adhaerens SVCTNB* (EDV23955.1), *Aplysia californica SVCTP* (XP_012935012.1) and *Acanthaster planci SVCT* (XP_022103910.1). The resulting datasets were then analysed as described above, using 1,000,000 generations and a burn-in of 2500. Convergence was achieved in all cases.

The phylogenies obtained for *Regucalcin* and *SVCT* were rooted using MEGA X [75], after conversion of the files from Nexus to Newick format using the application available at http://phylogeny.lirmm.fr/phylo_cgi/data_converter.cgi.

### Positively selected amino acid sites inference

Positively selected amino acid sites (PSS) were inferred using both codeML [76] as implemented in ADOPS [74] and FUBAR [77] as implemented in DataMonkey [78]. Gene sequence sets were defined based on the *Regucalcin* Bayesian phylogenies. Only sets with a minimum of five species were considered. The considered FASTA files represented the Sophophora *Regucalcin*, Sophophora *Dca*, Culicidae *Cu3*, Apoidea *Hy2*, Formicoidea *Hy1*, Lepidoptera *L2*, *L3* and *L4*, Ciprinidae *C1.2*, Teleost_w/o_Cyprinidae_Salmonidae and the Reptilia and Testudines, Aves and Mammalia *R1* and *R2* genes. codeML did not produce a valid output for the Aves *R1*, Mammalia *R2* and Teleost_w/o_Cyprinidae_Salmonidae datasets due to an unknown internal error that persisted after several trials. The results of the remaining datasets were deposited in the B + database (bpositive.i3s.up.pt; “The evolution of regucalcin-like genes” (BP2018000005)), which allows for the visualization and download of the project by any user [79, 80]. Inferred PSS with a probability equal or higher than 90% identified by both approaches, were considered as positive. In the Aves *R1*, Mammalia *R2* and Teleost_w/o_Cyprinidae_Salmonidae datasets, only the FUBAR results were considered. For each dataset, the PSS were marked on a 3D model structure prediction of a representative protein sequence, obtained using I-TASSER [81–83].

### *D. melanogaster Regucalcin* and *SVCT* interactome analyses

Protein-protein interactions (PPI) were obtained from EvoPPI 1.0 web tool [62], a platform that compiles all PPI reported in 12 databases. Searches were performed by selecting the appropriate species under the “Same species” query, and by using all available interactome databases. Only direct interactions (Interaction level 1) were considered. The queried gene IDs are for *D. melanogaster*: 32165 (*Regucalcin*), 41786 (*Dca*; there are no reported interactions), and 41259 (*SVCTP*); for *M. musculus*: 19733 (*Regucalcin*), 20522 (*SVCT1)*, 54338 (*SVCT2*) and 22626 (*SVCT3*); for *H. sapiens*: 9104 (*Regucalcin*), 9963 (*SVCT1*), 9962 (*SVCT2*) and 151295 (*SVCT3*).

The gene IDs of the *D. melanogaster* interactors were processed using the DRSC Integrative Ortholog Prediction Tool [84] in order to determine orthologous genes in *M. musculus* and *H. sapiens*. Genes with low confidence score or without any hit were analysed using a BLASTP reciprocal best hit approach [85] using the NCBI BLAST web interface (https://blast.ncbi.nlm.nih.gov/Blast.cgi#). The selected word size was three and low complexity regions were excluded.

### Regucalcin and Dca protein docking inferences

Protein docking inferences were performed using an in silico protocol available in the literature [86]. The *D. melanogaster* protein sequences were downloaded from NCBI as FASTA formatted files (accession numbers AAN09306.2 (Regucalcin), AGB95961.1 (Dca), NP_608711.3 (Histidine triad nucleotide binding protein 1), NP_476735.1 (Superoxide dismutase 1), and NP_732311.1 (14-3-3epsilon)).

### *D. melanogaster* RNAi experiments

The *Actin5C* GAL4 driver stock (25374) was obtained from the Bloomington Drosophila Stock Center (https://bdsc.indiana.edu), while the RNAi stocks for *Regucalcin* (105509) and *Dca* (103377) were supplied by the Vienna Drosophila Resource Center (https://stockcenter.vdrc.at). Fly stocks were kept at environmental chambers with a constant temperature of 25ºC and 12 h day/night cycles. Flies were reared on cornmeal food supplemented with yeast extract and depleted of VC. The driver/RNAi crosses were performed in both directions (♂ driver x ♀ RNAi; ♀ driver x ♂ RNAi) using 6 individuals of each stock per cross, using cornmeal food vials kept at 25ºC and 12 h day/night cycles. The progenitors were subsequently transferred to new vials every two days to maximize sample acquisition, until six vials were gathered. At the day of birth, the progeny was sorted according to the phenotype and gender.

## Supplementary information

**Additional file 1.** *Regucalcin and Dca D.* melanogaster RNAi crosses (**Table S1**), Regucalcin interactome information (**Table S2**), Regucalcin-like positively selected amino acid sites (**Table S3**), SVCT interactome information (**Table S4**), details on sequence data set preparation for phylogenetic analyses (**Table S5**), Regucalcin phylogenetic analyses (**Figures S1–S11**), distribution of Regucalcin predicted interacting residues (**Figures S12–S13**), and SVCT-like (**Figures S14–S22**) phylogenetic analyses.

## Data Availability

The FASTA files used for phylogenetic analyses, the PDB files containing the inferred structures and the Newick tree files are provided in Zenodo (10.5281/zenodo.6518249). Positively selected amino acid inferences are publicly available at B + database (http://bpositive.i3s.up.pt) under project BP2018000005.
