# Supplementary material for: The evolution of vitamin C biosynthesis and transport in animals
Source: BMC Ecol Evol. 2022 Jun 25;22:84. doi: 10.1186/s12862-022-02040-7 (PMC9233358; doi:10.1186/s12862-022-02040-7)
Supplement: Supplementary file 1 — Additional file 1. Regucalcin and Dca D. melanogaster RNAi crosses (Table S1), Regucalcin interactome information (Table S2), Regucalcin-like positively selected amino acid sites (Table S3), SVCT interactome information (Table S4), details on sequence data set preparation for phylogenetic analyses (Table S5), Regucalcin phylogenetic analyses (Figures S1–S11), distribution of Regucalcin predicted interacting residues (Figures S12–S13), and SVCT-like (Figures S14–S22) phylogenetic analyses. [file 12862_2022_2040_MOESM1_ESM.pdf]

The evolution of vitamin C biosynthesis and transport in animals

Pedro Duque, Cristina P. Vieira, Bárbara Bastos, Jorge Vieira

Additional tables

Additional file 1: Table S1 – *Regucalcin* and *Dca D. melanogaster* RNAi crosses progeny count.
Additional file 1: Table S2 – Conserved *Regucalcin* interactors between *D. melanogaster* and *H. sapiens*/*M. musculus*.
Additional file 1: Table S3 – PSS located outside of the active site and lid regions relative to the dataset's reference protein structures.
Additional file 1: Table S4 – Retrieved *D. melanogaster* SVCT interactome and corresponding orthologs in *H. sapiens*, as well as the current SVCT1, SVCT2 and SVCT3 *H. sapiens* interactomes.
Additional file 1: Table S5 – *Regucalcin* and *SVCT* sequences removed during dataset processing.

Additional figures

Additional file 1: Fig. S1 – Non-Bilaterian *Regucalcin* original (A) and collapsed (B) Bayesian phylogenies. In A, the species relevant taxonomic groups are represented next to the corresponding sequences and the different taxonomic groups are highlighted with distinct colours, following the colour scheme presented below the consensus tree. The outgroup sequence is not highlighted and can be seen at the bottom of the phylogeny. The proposed gene clusters after manual curation can be observed in B.
Additional file 1: Fig. S2 – Hemipteroid Assemblage and Blattodea *Regucalcin* original (A) and collapsed (B) Bayesian phylogenies. In A, the species relevant taxonomic groups are represented next to the corresponding sequences and the different taxonomic groups are highlighted with distinct colours, following the colour scheme presented below the consensus tree. The general outgroup sequence is not highlighted and can be seen at the bottom of the phylogeny, while the Remaining Insecta representative sequence is marked in light purple. The proposed gene clusters after manual curation can be observed in B.
Additional file 1: Fig. S3 – Coleoptera *Regucalcin* original (A) and collapsed (B) Bayesian phylogenies. In A, the species relevant taxonomic groups are represented next to the corresponding sequences and the different taxonomic groups are highlighted with distinct colours, following the colour scheme presented below the consensus tree. The outgroup sequence is not highlighted and can be seen at the bottom of the phylogeny The proposed gene clusters after manual curation can be observed in B.
Additional file 1: Fig. S4 – Hymenoptera *Regucalcin* original (A) and collapsed (B) Bayesian phylogenies. In A, the species relevant taxonomic groups are represented next to the corresponding sequences and the different taxonomic groups are highlighted with distinct colours, following the colour scheme presented below the consensus tree. The outgroup sequence is not highlighted and can be seen at the bottom of the phylogeny The proposed gene clusters after manual curation can be observed in B.
Additional file 1: Fig. S5 – Diptera *Regucalcin* original (A) and collapsed (B) Bayesian phylogenies. In A, the species relevant taxonomic groups are represented next to the corresponding sequences and the different taxonomic groups are highlighted with distinct colours, following the colour scheme presented below the consensus tree. The outgroup sequence is not highlighted and can be seen at the bottom of the phylogeny The proposed gene clusters after manual curation can be observed in B.
Additional file 1: Fig. S6 – Lepidoptera *Regucalcin* original (A) and collapsed (B) Bayesian phylogenies. In A, the species relevant taxonomic groups are represented next to the corresponding sequences and the different taxonomic groups are highlighted with distinct colours, following the colour scheme presented below the consensus tree. The outgroup sequence is not highlighted and can be seen at the bottom of the phylogeny The proposed gene clusters after manual curation can be observed in B.
Additional file 1: Fig. S7 – Proposed *Regucalcin e*volutionary scenario across the Insecta. *Regucalcin* presence is highlighted in green, absence in red and uncertainty regarding gene loss/presence in light blue. Gene duplications affecting a single species of a genus are marked with a *, while those which affected two or more species from the same genus are marked with a #. The number of species present in the final dataset and total number of species analysed for each taxonomic group can be seen within parentheses, respectively. Taxonomic relationships are depicted as seen in literature [1-10].
Additional file 1: Fig. S8 – Non-Lophotrochozoa *Regucalcin* original (A) and collapsed (B) Bayesian phylogenies. In A, the species relevant taxonomic groups are represented next to the corresponding sequences and the different taxonomic groups are highlighted with distinct colours, following the colour scheme presented below the consensus tree. The general outgroup sequence is not highlighted and can be seen at the bottom of the phylogeny, while the Insecta representative sequence is marked in light pink. The proposed gene clusters after manual curation can be observed in B.
Additional file 1: Fig. S9 – Lophotrochozoa *Regucalcin* original (A) and collapsed (B) Bayesian phylogenies. In A, the species relevant taxonomic groups are represented next to the corresponding sequences and the different taxonomic groups are highlighted with distinct colours, following the colour scheme presented below the consensus tree. The outgroup sequence is not highlighted and can be seen at the bottom of the phylogeny The proposed gene clusters after manual curation can be observed in B.
Additional file 1: Fig. S10 – Basal deuterostomians *Regucalcin* original (A) and collapsed (B) Bayesian phylogenies. In A, the species relevant taxonomic groups are represented next to the corresponding sequences and the different taxonomic groups are highlighted with distinct colours, following the colour scheme presented below the consensus tree. The outgroup sequence is not highlighted and can be seen at the bottom of the phylogeny The proposed gene clusters after manual curation can be observed in B.
Additional file 1: Fig. S11 – Deuterostomians *Regucalcin* original (A) and collapsed (B) Bayesian phylogenies. In A, the species relevant taxonomic groups are represented next to the corresponding sequences and the different taxonomic groups are highlighted with distinct colours, following the colour scheme presented below the consensus tree. The outgroup sequence is not highlighted and can be seen at the top of the phylogeny The proposed gene clusters after manual curation can be observed in B.
Additional file 1: Fig. S12 – Distribution of predicted interacting residues on the *D. melanogaster* Regucalcin AAN09306.2 (A) and Dca AGB95961.1 (B) relative to the 14-3-3 protein epsilon (NP_033562.3), Histidine triad nucleotide-binding protein 1 (NP_005331.1) and Superoxide dismutase 1 (NP_000445.1) models. The Regucalcin and Dca predicted structutes are highlighted in cyan, while the remaining protein models are represented in green. The interacting residues can be seen in red. The represented interaction models are the ones with the highest Regucalcin and Dca interacting residues within the lowest Z-score cluster provided by HADDOCK. For comparison purposes, all the Regucalcin and Dca structures have the lid region on the top portion of the panels. This figure allows the visualization of the distinct overall interaction surfaces concerning both proteins.
Additional file 1: Fig. S13 – Distribution of predicted interacting residues on the human Regucalcin (RCSB PDB accession number 3G4E) relative to the human 14-3-3 protein epsilon (3UAL), Histidine triad nucleotide-binding protein 1 (6B42) and Superoxide dismutase 1 (6FLH) crystal structures. The Regucalcin structute is highlighted in cyan, while the remaining proteins are represented in green. The interacting residues can be seen in red. The represented interaction models are the ones with the highest Regucalcin interacting residues within the lowest Z-score cluster provided by HADDOCK. For comparison purposes, the Regucalcin structure has the lid region on the top portion of the panels. This figure allows the visualization of an interaction surface overall very similiar to the one observed in the *D. melanogaster* Regucalcin (AAN09306.2), indicating that the distinct interacting surface that emerged in the *D. melanogaster* Dca (AGB95961.1) is likely the consequence of gene neofunctionalization processes after duplication.
Additional file 1: Fig. S14 – Non-bilaterian *Sodium-dependent Vitamin C transporter (SVCTNB)* original (A) and collapsed (B) Bayesian phylogenies. In A, the species relevant taxonomic groups are represented next to the corresponding sequences and the different taxonomic groups are highlighted with distinct colours, following the colour scheme presented below the consensus tree. The three outgroup sequences are not highlighted and can be seen at the top of the phylogeny. The proposed gene clusters after manual curation can be observed in B.
Additional file 1: Fig. S15 – Non-Lophotrochozoa *Sodium-dependent Vitamin C transporter (SVCTP NL)* original (A) and collapsed (B) Bayesian phylogenies. In A, the species relevant taxonomic groups are represented next to the corresponding sequences and the different taxonomic groups are highlighted with distinct colours, following the colour scheme presented below the consensus tree. The three outgroup sequences are not highlighted and can be seen at the bottom of the phylogeny. The proposed gene clusters after manual curation can be observed in B.
Additional file 1: Fig. S16 – Lophotrochozoa *Sodium-dependent Vitamin C transporter (SVCTP L)* original (A) and collapsed (B) Bayesian phylogenies. In A, the species relevant taxonomic groups are represented next to the corresponding sequences and the different taxonomic groups are highlighted with distinct colours, following the colour scheme presented below the consensus tree. The three outgroup sequences are not highlighted and can be seen at the bottom of the phylogeny. The proposed gene clusters after manual curation can be observed in B.
Additional file 1: Fig. S17 – Basal deuterostomian *Sodium-dependent Vitamin C transporter (SVCT BD)* original (A) and collapsed (B) Bayesian phylogenies. In A, the species relevant taxonomic groups are represented next to the corresponding sequences and the different taxonomic groups are highlighted with distinct colours, following the colour scheme presented below the consensus tree. The seven outgroup sequences are not highlighted and can be seen spread across the phylogeny. The proposed gene clusters after manual curation can be observed in B.
Additional file 1: Fig. S18 – *Sodium-dependent Vitamin C transporter 1* (*SVCT1*) original (A) and collapsed (B) Bayesian phylogenies. In A, the species relevant taxonomic groups are represented next to the corresponding sequences and the different taxonomic groups are highlighted with distinct colours, following the colour scheme presented below the consensus tree. The four outgroup sequences are not highlighted and can be seen at the top of the phylogeny. The proposed gene clusters after manual curation can be observed in B.
Additional file 1: Fig. S19 – *Sodium-dependent Vitamin C transporter 2* (*SVCT2*) original (A) and collapsed (B) Bayesian phylogenies. In A, the species relevant taxonomic groups are represented next to the corresponding sequences and the different taxonomic groups are highlighted with distinct colours, following the colour scheme presented below the consensus tree. The four outgroup sequences are not highlighted, and can be seen at the top and bottom of the phylogeny. The proposed gene clusters after manual curation can be observed in B.
Additional file 1: Fig. S20 – *Sodium-dependent Vitamin C transporter 3* (*SVCT3*) original (A) and collapsed (B) Bayesian phylogenies. In A, the species relevant taxonomic groups are represented next to the corresponding sequences and the different taxonomic groups are highlighted with distinct colours, following the colour scheme presented below the consensus tree. The four outgroup sequences are not highlighted, and can be seen at the top and bottom of the phylogeny. The proposed gene clusters after manual curation can be observed in B.
Additional file 1: Fig. S21 – *Sodium-dependent Vitamin C transporter 4* (*SVCT4*) original (A) and collapsed (B) Bayesian phylogenies. In A, the species relevant taxonomic groups are represented next to the corresponding sequences and the different taxonomic groups are highlighted with distinct colours, following the colour scheme presented below the consensus tree. The four outgroup sequences are not highlighted, and can be seen at the top of the phylogeny.. The proposed gene clusters after manual curation can be observed in B.
Additional file 1: Fig. S22 – Putative *Sodium-dependent Vitamin C transporter 5* (*SVCT5*) original (A) and collapsed (B) Bayesian phylogenies. In A, the species relevant taxonomic groups are represented next to the corresponding sequences and the different taxonomic groups are highlighted with distinct colours, following the colour scheme presented below the consensus tree. The 16 outgroup sequences are not highlighted and can be seen at the top of the phylogeny. The proposed gene clusters after manual curation can be observed in B.

References

1. Maddison DR, Schulz K-S, Maddison WP. The Tree of Life Web Project*. Zootaxa. 2007;1668(1):19-40.
2. van der Linde K, Houle D, Spicer GS, Steppan SJ. A supermatrix-based molecular phylogeny of the family Drosophilidae. Genet Res (Camb). 2010;92(1):25-38.
3. Wiegmann BM, Trautwein MD, Winkler IS, Barr NB, Kim J-W, Lambkin C, et al. Episodic radiations in the fly tree of life. Proceedings of the National Academy of Sciences. 2011;108(14):5690.
4. Song N, Liang A-P, Bu C-P. A Molecular Phylogeny of Hemiptera Inferred from Mitochondrial Genome Sequences. PloS one. 2012;7(11):e48778.
5. Song F, Li H, Jiang P, Zhou X, Liu J, Sun C, et al. Capturing the Phylogeny of Holometabola with Mitochondrial Genome Data and Bayesian Site-Heterogeneous Mixture Models. Genome Biol Evol. 2016;8(5):1411-26.
6. Li H, Leavengood JM, Jr., Chapman EG, Burkhardt D, Song F, Jiang P, et al. Mitochondrial phylogenomics of Hemiptera reveals adaptive innovations driving the diversification of true bugs. Proceedings Biological sciences. 2017;284(1862):20171223.
7. Peters RS, Krogmann L, Mayer C, Donath A, Gunkel S, Meusemann K, et al. Evolutionary History of the Hymenoptera. Current biology : CB. 2017;27(7):1013-8.
8. Johnson Kevin P, Dietrich Christopher H, Friedrich F, Beutel Rolf G, Wipfler B, Peters Ralph S, et al. Phylogenomics and the evolution of hemipteroid insects. Proceedings of the National Academy of Sciences. 2018;115(50):12775-80.
9. O'Grady PM, DeSalle R. Phylogeny of the Genus Drosophila. Genetics. 2018;209(1):1-25.
10. Zhang S-Q, Che L-H, Li Y, Dan L, Pang H, Ślipiński A, et al. Evolutionary history of Coleoptera revealed by extensive sampling of genes and species. Nature Communications. 2018;9(1):205.

Additional file 1: Table S1

Crossing	Straight ♀	Curly ♀	Straight ♂	Curly ♂	X^2 ♀	X^2 ♂
RGN (#105509) ♂ x *Act5C/GAL4* (#25374) ♀	40	445	11	454	338,19 (P<0,001)	422,04 (P<0,001)
RGN (#105509) ♀ x *Act5C/GAL4* (#25374) ♂	81	407	3	355	217,78 (P<0,001)	346,10 (P<0,001)
Dca (#103377) ♂ x *Act5C/GAL4* (#25374) ♀	70	324	16	303	163,65 (P<0,001)	258,21 (P<0,001)
Dca (#103377) ♀ x *Act5C/GAL4* (#25374) ♂	56	194	25	159	76,176 (P<0,001)	97,59 (P<0,001)

Additional file 1: Table S2

D. melanogaster **Regucalcin interactors**	**Interactor homologs (*H. sapiens*)**	**Interactor homologs (*M. musculus*)**
33471	3094	n.a.
39251	6647	n.a.
39476	3336	n.a.
39505	9948	n.a.
42186	n.a.	22627

Additional file 1: Table S3

Dataset	Protein reference PSS
Sophophora Regucalcin	I^{8}, A^{9}
Sophophora Dca	L^{108}, S^{157}, S^{197}
Apoidea Hy2	T^{47}, A^{91}, L^{149}
Formicoidea Hy1	A^{17}, V^{100}, R^{250}, T^{312}
Lepidoptera L2	Q^{305}
Teleost without Salmonidae and Cyprinidae	A^{181}
Reptilia R1	D^{189}
Aves R1	V^{46}
Aves R2	Y^{14}, R^{15}, A^{45}, G^{47}, S^{86}, A^{91}, K^{195}, V^{281}
Mammalia R2	Q^{48}, A^{87}, N^{95}

Additional file 1:Table S4

D. melanogaster SVCT Interactome	*H. sapiens* orthologs	SVCT1 Interactome	SVCT2 Interactome	SVCT3 Interactome
32179	No ortholog retrived (BLAST + DIOPT)	2885	4734	3480
32630	9972	3198	7316	5625
32851	129401	3881	10555	
34292	Low confidence hits	4223	27065	
35226	7274	4690	55238	
35305	6741	5295	55744	
35664	161424	5335	79669	
36491	55755	10768	79956	
37637	54940	23281	84522	
38045	27044	30008	85025	
39382	65095	54507	254428	
40233	8755	81851		
40532	No ortholog retrived (BLAST + DIOPT)	83900		
40806	152007	85291		
41270	56915	125115		
41360	1743	375791		
42453	1017	386675		
43057	No ortholog retrived (BLAST + DIOPT)	386676		
2768852	4140	386677		
		386680		
		386681		
		386682		
		386683		
		388677		
		653240		

Additional file 1:Table S5

Isoforms removed		Redundant sequences from different species	Problematic Sequences Removed	
Regucalcin	SVCT	Regucalcin	Regucalcin	SVCT
Pogona_vitticeps_XP_020652482.1	Acanthaster_planci_(crown-of-thorns_starfish)_Acanthasteridae_Asteroidea_XP_022080025.1	Haliaeetus_albicilla_(white-tailed_eagle)_Accipitridae_XP_009920517.1 replaces the following sequences:	Bactrocera_latifrons_XP_018802379.1	Acanthisitta_chloris_rifleman_Acanthisittidae_Aves_XP_009075854.1
Manacus_vitellinus_XP_008930214.2	Acinonyx_jubatus_(cheetah)_Felidae_Mammalia_XP_014928547.1/XP_014928548.1	Haliaeetus_leucocephalus_(bald_eagle)_Accipitridae_XP_010578757.1	Bemisia_tabaci_XP_018902740.1	Aedes_aegypti_yellow_fever_mosquito_Culicidae_Insecta_EJY57974.1
Tupaia_chinensis_ELV14210.1	Ailuropoda_melanoleuca_(giant_panda)_Ursidae_Mammalia_XP_011233547.1/XP_019661063.1	Haliaeetus_albicilla_(white-tailed_eagle)_Accipitridae_KFQ06724.1	Branchiostoma_floridae_EEN58249.1	Aedes_albopictus_Asian_tiger_mosquito_Culicidae_Insecta_XP_019541343.1
Myotis_brandtii_XP_014398972.1	Alligator_mississippiensis_(American_alligator)_Alligatoridae_KYO34297.1/XP_019337984.1/XP_019346970.1		Gallus_gallus_XP_015156358.1	Anolis_carolinensis_green_anole_Dactyloidae_XP_008108299.1
Folsomia_candida_XP_021956083.1	Anolis_carolinensis_(green_anole)_Dactyloidae_XP_008109011.1	Corvus_brachyrhynchos_(American_crow)_Corvidae_XP_008627561.1 replaces the following sequences:	Nilaparvata_lugens_XP_022187325.1	Aplysia_californica_California_sea_hare_Aplysiidae_Gastropoda_XP_005089904.1
Ailuropoda_melanoleuca_EFB16351.1	Aotus_nancymaae_(Ma's_night_monkey)_Aotidae_Mammalia_XP_012301315.1/XP_012329553.1	Corvus_brachyrhynchos_(American_crow)_Corvidae_XP_017584063.1	Nilaparvata_lugens_XP_022203265.1	Aplysia_californica_California_sea_hare_Aplysiidae_Gastropoda_XP_005089905.2
Cricetulus_griseus_XP_007633712.1/XP_007629332.1	Balaenoptera_acutorostrata_scammoni_(minke_whale)_Balaenopteridae_Mammalia_XP_007188011.1/XP_007188012.1/XP_007191768.1	Corvus_cornix_cornix_(hooded_crow)_Corvidae_XP_019138371.1	Oikopleura_dioica_CBY20807.1	Apostichopus_japonicus_Japanese_sea_cucumber_Stichopodidae_Holothuroidea_PIK43178.1
Phascolarctos_cinereus_XP_020848583.1	Bison_bison_bison_(American_bison)_Bovidae_Mammalia_XP_010848622.1/XP_010859518.1/XP_010859519.1	Corvus_cornix_cornix_(hooded_crow)_Corvidae_XP_019138372.1	Peromyscus_maniculatus_bairdii_XP_015844817.1	Atta_colombica_ants_Formicidae_Insecta_KYM76322.1
Charadrius_vociferus_XP_009880409.1	Bos_indicus_(zebu_cattle)_Bovidae_Mammalia_XP_019820577.1/XP_019820578.1	Corvus_cornix_cornix_(hooded_crow)_Corvidae_XP_019138373.1	Pogona_vitticeps_XP_020652480.1	Balearica_regulorum_gibbericeps_East_African_grey_crowned_crane_Gruidae_Aves_XP_010295977.1
Opisthocomus_hoazin_XP_009929456.1/XP_009929455.1	Bos_mutus_(wild_yak)_Bovidae_Mammalia_XP_005889734.1	Corvus_brachyrhynchos_(American_crow)_Corvidae_KFO55830.1	Trichomalopsis_sarcophagae_OXU28709.1	Bos_indicus_zebu_cattle_Bovidae_Mammalia_XP_019820576.1
Calypte_anna_XP_008496314.1	Bos_taurus_(cattle)_Bovidae_Mammalia_DAA30365.1/DAA32438.1/XP_010800643.1/XP_010805675.1/XP_010805677.1		Tupaia_chinensis_XP_006164595.1	Bubalus_bubalis_water_buffalo_Bovidae_Mammalia_XP_006059070.1
Lonchura_striata_domestica_XP_021402306.1	Branchiostoma_belcheri_(Belcher's_lancelet)_Branchiostomidae_XP_019616280.1/XP_019633777.1/XP_019636192.1	Ovis_aries_(sheep)_Bovidae_XP_011961489.1 replaces the following sequences:		Cebus_capucinus_imitator_white_faced_sapajou_Cebidae_Mammalia_XP_017390574.1
Peromyscus_maniculatus_bairdii_XP_015844818.1/XP_015844821.1/XP_015844822.1	Bubalus_bubalis_(water_buffalo)_Bovidae_Mammalia_XP_006059067.1/XP_006070876.1/XP_006059069.1/XP_006070874.1/XP_006070881.1/XP_006070882.1	Ovis_aries_(sheep)_Bovidae_XP_011961491.1		Cercocebus_atys_sooty_mangabey_Cercopithecidae_Mammalia_XP_011918261.1
Cyprinus_carpio_KTF96356.1	Callithrix_jacchus_(white-tufted-ear_marmoset)_Cebidae_Mammalia_XP_002749849.1/XP_008983951.1	Ovis_aries_(sheep)_Bovidae_XP_011961487.1		Cercocebus_atys_sooty_mangabey_Cercopithecidae_Mammalia_XP_011945825.1
Pygocentrus_nattereri_XP_017557673.1	Callorhinchus_milii_(elephant_shark)_Callorhinchidae_Chondrichthyes_XP_007903283.1/XP_007904442.1/XP_007904444.1	Ovis_aries_(sheep)_Bovidae_XP_011961488.1		Cervus_elaphus_hippelaphus_red_deer_Cervidae_Mammalia_OWK13001.1
Strongylocentrotus_purpuratus_XP_011676044.1	Camelus_bactrianus_(Bactrian_camel)_Camelidae_Mammalia_XP_010945808.1/XP_010947100.1	Ovis_aries_(sheep)_Bovidae_XP_011961492.1		Chaetura_pelagica_chimney_swift_Apodidae_Aves_XP_009994567.1
Nilaparvata_lugens_XP_022204098.1	Camelus_ferus_(Wild_Bactrian_camel)_Camelidae_Mammalia_XP_014416507.1	Ovis_aries_(sheep)_Bovidae_NP_001124407.1		Crassostrea_virginica_eastern_oyster_Ostreidae_Bivalvia_XP_022290101.1
Acanthaster_planci_XP_022089748.1	Canis_lupus_familiaris_(dog)_Canidae_Mammalia_NP_001103426.2/XP_013966522.1	Ovis_aries_musimon_(mouflon)_Bovidae_NP_001124407.1		Crassostrea_virginica_eastern_oyster_Ostreidae_Bivalvia_XP_022290103.1
Papilio_xuthus_XP_013166227.1	Capra_hircus_(goat)_Bovidae_Mammalia_XP_013818972.2/XP_013821053.1			Crassostrea_virginica_eastern_oyster_Ostreidae_Bivalvia_XP_022343879.1
Varroa_destructor_XP_022650147.1	Carlito_syrichta_(Philippine_tarsier)_Tarsiidae_Mammalia_XP_008057056.1			Crassostrea_virginica_eastern_oyster_Ostreidae_Bivalvia_XP_022344084.1
Folsomia_candida_OXA48477.1/OXA46847.1/XP_021965762.1/OXA40016.1	Cavia_porcellus_(domestic_guinea_pig)_Caviidae_Mammalia_XP_012996433.1			Cyprinus_carpio_common_carp_Cyprinidae_Actinopteri_KTF84040.1
Zootermopsis_nevadensis_XP_021924408.1	Ceratotherium_simum_simum_(southern_white_rhinoceros)_Rhinocerotidae_Mammalia_XP_004430790.2			Cyprinus_carpio_common_carp_Cyprinidae_Actinopteri_XP_018944039.1
Crassostrea_gigas_EKC25142.1	Cercocebus_atys_(sooty_mangabey)_Cercopithecidae_Mammalia_XP_011918257.1/XP_011918258.1			Cyprinus_carpio_common_carp_Cyprinidae_Actinopteri_XP_018962645.1
Diachasma_alloeum_XP_015116164.1/XP_015110079.1	Chelonia_mydas_(green_sea_turtle)_Cheloniidae_EMP30536.1/XP_007059233.1			Diploscapter_pachys_nematodes_Diploscapteridae_Chromadorea_PAV82999.1
Fopius_arisanus_XP_011306302.1	Chinchilla_lanigera_(long-tailed_chinchilla)_Chinchillidae_Mammalia_XP_005382134.1/XP_005405541.1/XP_005405542.1			Equus_caballus_horse_Equidae_Mammalia_XP_014595792.1
Microplitis_demolitor_XP_008547468.1/XP_008547469.1	Chlorocebus_sabaeus_(green_monkey)_Cercopithecidae_Mammalia_XP_008012810.1/XP_008017163.1			Felis_catus_domestic_cat_Felidae_Mammalia_XP_019688440.1
Bombyx_mori_XP_021206751.1/XP_004930716.1	Chrysochloris_asiatica_(Cape_golden_mole)_Chrysochloridae_Mammalia_XP_006866196.1			Gallus_gallus_chicken_Phasianidae_Aves_XP_015145566.1
Papilio_xuthus_XP_013166225.1	Colobus_angolensis_palliatus_(Angolan_colobus)_Cercopithecidae_Mammalia_XP_011787622.1			Geospiza_fortis_medium_ground_finch_Thraupidae_Aves_XP_005421727.1
Drosophila_pseudoobscura_pseudoobscura_KRS99979.1	Condylura_cristata_(star-nosed_mole)_Talpidae_Mammalia_XP_004686845.1			Geospiza_fortis_medium_ground_finch_Thraupidae_Aves_XP_014166690.1
Dendroctonus_ponderosae_ENN81654.1	Coturnix_japonica_(Japanese_quail)_Phasianidae_Aves_XP_015731501.1/XP_015731502.1			Homo_sapiens_human_Hominidae_Mammalia_XP_011542067.1
Nothobranchius_furzeri_XP_015807699.1	Crassostrea_gigas_(Pacific_oyster)_Ostreidae_Bivalvia_XP_011424638.1/XP_011424641.1/XP_011428055.1/XP_019927372.1			Jaculus_jaculus_lesser_Egyptian_jerboa_Dipodidae_Mammalia_XP_004662891.2
Oreochromis_niloticus_XP_005466161.1	Crassostrea_virginica_(eastern_oyster)_Ostreidae_Bivalvia_XP_022286055.1/XP_022290098.1/XP_022333560.1/XP_022333561.1/XP_022333562.1/XP_022340458.1/XP_022341469.1/XP_022342795.1/XP_022342796.1/XP_022344082.1/XP_022344085.1			Lates_calcarifer_barramundi_perch_Centropomidae_Actinopteri_XP_018551782.1
Eufriesea_mexicana_OAD58557.1	Cricetulus_griseus_(Chinese_hamster)_Cricetidae_Mammalia_XP_003507645.1/XP_007609110.1/XP_007614257.1/XP_007646065.1			Lepisosteus_oculatus_spotted_gar_Lepisosteidae_Actinopteri_XP_015205203.1
Trachymyrmex_cornetzi_XP_018374114.1	Crocodylus_porosus_(Australian_saltwater_crocodile)_Crocodylidae_XP_019401496.1			Limulus_polyphemus_Atlantic_horseshoe_crab_Limulidae_Merostomata_XP_022237907.1
Harpegnathos_saltator_XP_011153703.1	Danio_rerio_(zebrafish)_Cyprinidae_Actinopteri_XP_021327808.1			Limulus_polyphemus_Atlantic_horseshoe_crab_Limulidae_Merostomata_XP_022242450.1
Ooceraea_biroi_XP_011332720.1	Delphinapterus_leucas_(beluga_whale)_Monodontidae_Mammalia_XP_022424023.1/XP_022444628.1			Lingula_anatina_brachiopods_Lingulidae_Lingulata_XP_013379940.1
Vollenhovia_emeryi_XP_011881380.1	Diploscapter_pachys_(nematodes)_Diploscapteridae_Chromadorea_PAV61857.1/PAV61859.1			Lingula_anatina_brachiopods_Lingulidae_Lingulata_XP_013420342.1
Bactrocera_oleae_XP_014103595.1	Dipodomys_ordii_(Ord's_kangaroo_rat)_Heteromyidae_Mammalia_XP_012866486.1			Macaca_mulatta_Rhesus_monkey_Cercopithecidae_Mammalia_EHH25877.1
Aedes_albopictus_XP_019529541.1	Echinops_telfairi_(small_Madagascar_hedgehog)_Tenrecidae_Mammalia_XP_004701672.1			Macaca_mulatta_Rhesus_monkey_Cercopithecidae_Mammalia_XP_014966596.1
Halyomorpha_halys_XP_014289311.1	Eptesicus_fuscus_(big_brown_bat)_Vespertilionidae_Mammalia_XP_008139890.1			Macaca_nemestrina_pig_tailed_macaque_Cercopithecidae_Mammalia_XP_011714694.1
Aedes_aegypti_EAT34066.1	Equus_asinus_(ass)_Equidae_Mammalia_XP_014694854.1			Manacus_vitellinus_golden_collared_manakin_Pipridae_Aves_XP_008918474.2
Aedes_albopictus_XP_019534284.1	Equus_przewalskii_(Przewalski's_horse)_Equidae_Mammalia_XP_008528175.1			Manis_javanica_Malayan_pangolin_Manidae_Mammalia_XP_017528582.1
Macrostomum_lignano_PAA52790.1	Erinaceus_europaeus_(western_European_hedgehog)_Erinaceidae_Mammalia_XP_007517602.1/XP_007520676.2			Melipona_quadrifasciata_bees_Apidae_Insecta_KOX68501.1
Drosophila_simulans_KMZ03555.1	Felis_catus_(domestic_cat)_Felidae_Mammalia_XP_011278903.2XP_019681421.1/XP_019682359.1/XP_019682360.1/XP_019688435.1/XP_019688438.1			Melopsittacus_undulatus_budgerigar_Psittaculidae_Aves_XP_012983231.1
Bombyx_mori_XP_021206751.1	Ficedula_albicollis_(collared_flycatcher)_Muscicapidae_Aves_XP_005053979.1/XP_005053980.1/XP_005053982.1/XP_016157219.1			Mizuhopecten_yessoensis_Yesso_scallop_Pectinidae_Bivalvia_XP_021348630.1
Agrilus_planipennis_XP_018333452.1/XP_018333449.1	Folsomia_candida_(springtails)_Isotomidae_Collembola_XP_021968646.1			Mizuhopecten_yessoensis_Yesso_scallop_Pectinidae_Bivalvia_XP_021369087.1
Folsomia_candida_XP_021965762.1	Fukomys_damarensis_(Damara_mole-rat)_Bathyergidae_Mammalia_XP_010610167.1/XP_010612094.1/XP_010614482.1/XP_010614484.1/XP_010614485.1			Mustela_putorius_furo_domestic_ferret_Mustelidae_Mammalia_XP_012917028.1
Crassostrea_gigas_XP_019926340.1	Fundulus_heteroclitus_(mummichog)_Fundulidae_Actinopteri_XP_021174252.1			Myotis_lucifugus_little_brown_bat_Vespertilionidae_Mammalia_XP_014309333.1
	Galeopterus_variegatus_(Sunda_flying_lemur)_Cynocephalidae_Mammalia_XP_008566841.1/XP_008566842.1			Octopus_bimaculoides_cephalopods_Octopodidae_Cephalopoda_KOF78689.1
	Gavialis_gangeticus_(Gharial)_Gavialidae_XP_019369887.1			Orbicella_faveolata_stony_corals_Merulinidae_Anthozoa_XP_020617303.1
	Gorilla_gorilla_gorilla_(western_lowland_gorilla)_Hominidae_Mammalia_XP_018883549.1			Ovis_aries_musimon_mouflon_Bovidae_Mammalia_XP_012006070.1
	Haplochromis_burtoni_(Burton's_mouthbrooder)_Cichlidae_Actinopteri_XP_005924413.1			Panthera_tigris_altaica_Amur_tiger_Felidae_Mammalia_XP_015391065.1
	Heterocephalus_glaber_(naked_mole-rat)_Bathyergidae_Mammalia_EHB12075.1/XP_004840833.1/XP_004841888.1			Patagioenas_fasciata_monilis_band_tailed_pigeon_Columbidae_Aves_OPJ86890.1
	Hippocampus_comes_(tiger_tail_seahorse)_Syngnathidae_Actinopteri_XP_019712019.1/XP_019742679.1/XP_019750397.1/XP_019750399.1			Pelodiscus_sinensis_Chinese_soft_shelled_turtle_Trionychidae_XP_006115849.1
	Homo_sapiens_(human)_Hominidae_Mammalia_NP_001138361.1/NP_976072.1/XP_005272206.3/XP_006714804.2			Priapulus_caudatus_priapulids_Priapulidae_Priapulimorpha_XP_014661635.1
	Ictidomys_tridecemlineatus_(thirteen-lined_ground_squirrel)_Sciuridae_Mammalia_XP_005330546.1/XP_021586823.1			Pteropus_vampyrus_large_flying_fox_Pteropodidae_Mammalia_XP_011380633.1
	Kryptolebias_marmoratus_(mangrove_rivulus)_Actinopteri_XP_017296453.1			Stylophora_pistillata_stony_corals_Pocilloporidae_Anthozoa_XP_022788733.1
	Larimichthys_crocea_(large_yellow_croaker)_Sciaenidae_Actinopteri_KKF20623.1/KKF22133.1			Tauraco_erythrolophus_red_crested_turaco_Musophagidae_Aves_XP_009987685.1
	Lates_calcarifer_(barramundi_perch)_Centropomidae_Actinopteri_XP_018548148.1			Tinamus_guttatus_white_throated_tinamou_Tinamidae_Aves_XP_010211208.1
	Lepidothrix_coronata_(blue-crowned_manakin)_Pipridae_Aves_XP_017685859.1/XP_017685861.1/XP_017685862.1			Tinamus_guttatus_white_throated_tinamou_Tinamidae_Aves_XP_010224796.1
	Lepisosteus_oculatus_(spotted_gar)_Lepisosteidae_Actinopteri_XP_006625701.2/XP_015197789.1			Tyto_alba_barn_owl_Tytonidae_Aves_XP_009964161.1
	Limulus_polyphemus_(Atlantic_horseshoe_crab)_Limulidae_Merostomata_XP_022237906.1			
	Lingula_anatina_(brachiopods)_Lingulidae_Lingulata_XP_013391986.1/XP_013391995.1/XP_013392007.1			
	Lipotes_vexillifer_(Yangtze_River_dolphin)_Lipotidae_Mammalia_XP_007445894.1			
	Macaca_fascicularis_(crab-eating_macaque)_Cercopithecidae_Mammalia_EHH55177.1/XP_005568465.1/XP_005574393.1/XP_005574394.1/XP_015288308.1/XP_015288310.1			
	Macaca_mulatta_(Rhesus_monkey)_Cercopithecidae_Mammalia_NP_001248123.1/XP_001097078.2			
	Macaca_nemestrina_(pig-tailed_macaque)_Cercopithecidae_Mammalia_XP_011738994.1/XP_011738995.1			
	Macrostomum_lignano_(flatworms)_Macrostomidae_PAA49348.1			
	Manacus_vitellinus_(golden-collared_manakin)_Pipridae_Aves_XP_008918474.1/XP_008921143.2			
	Mandrillus_leucophaeus_(drill)_Cercopithecidae_Mammalia_XP_011820016.1			
	Manis_javanica_(Malayan_pangolin)_Manidae_Mammalia_XP_017528580.1/XP_017528581.1/XP_017534207.1/XP_017534208.1/XP_017534209.1			
	Marmota_marmota_marmota_(Alpine_marmot)_Sciuridae_Mammalia_XP_015336892.1			
	Maylandia_zebra_(zebra_mbuna)_Cichlidae_Actinopteri_XP_004547103.1			
	Mesocricetus_auratus_(golden_hamster)_Cricetidae_Mammalia_XP_021089937.1			
	Microcebus_murinus_(gray mouse lemur)_Cheirogaleidae_Mammalia_XP_012595665.1/XP_012600205.1/XP_012604476.1/XP_012610427.1/XP_020138019.1/XP_012595665.1/XP_012600205.1/XP_012604476.1/XP_012610427.1/XP_020138019.1			
	Microtus_ochrogaster_(prairie_vole)_Cricetidae_Mammalia_XP_005361666.1			
	Miniopterus_natalensis_(bats)_Vespertilionidae_Mammalia_XP_016059225.1			
	Mizuhopecten_yessoensis_(Yesso_scallop)_Pectinidae_Bivalvia_OWF42062.1/OWF42063.1/XP_021361216.1/XP_021369108.1/XP_021369664.1/XP_021372140.1			
	Monodelphis_domestica_(gray_short-tailed_opossum)_Didelphidae_Mammalia_XP_007501759.1			
	Mustela_putorius_furo_(domestic_ferret)_Mustelidae_Mammalia_XP_004741767.1/XP_004741768.1/XP_012907893.1/XP_012907895.1/XP_012917027.1/XP_012917029.1			
	Myotis_brandtii_(Brandt's_bat)_Vespertilionidae_Mammalia_EPQ12979.1/XP_014390630.1			
	Myotis_davidii_(bats)_Vespertilionidae_Mammalia_XP_006756759.1			
	Myotis_lucifugus_(little_brown_bat)_Vespertilionidae_Mammalia_XP_006086584.1			
	Nannospalax_galili_(Upper_Galilee_mountains_blind_mole_rat)_Spalacidae_Mammalia_XP_008823967.1			
	Nomascus_leucogenys_(northern_white-cheeked_gibbon)_Hylobatidae_Mammalia_XP_003272433.1			
	Notothenia_coriiceps_(black_rockcod)_Nototheniidae_Actinopteri_XP_010776876.1			
	Numida_meleagris_(helmeted_guineafowl)_Numididae_Aves_XP_021254340.1/XP_021266378.1/XP_021266379.1/XP_021266382.1			
	Odobenus_rosmarus_divergens_(Pacific_walrus)_Odobenidae_Mammalia_XP_012416959.1			
	Oncorhynchus_kisutch_(coho_salmon)_Salmonidae_Actinopteri_XP_020336643.1/XP_020336644.1			
	Oncorhynchus_mykiss_(rainbow_trout)_Salmonidae_Actinopteri_CDQ66965.1			
	Orbicella_faveolata_(stony_corals)_Merulinidae_Anthozoa_XP_020615395.1/XP_020617462.1			
	Orcinus_orca_(killer_whale)_Delphinidae_Mammalia_XP_004262747.1/XP_004280352.1			
	Oreochromis_niloticus_(Nile_tilapia)_Cichlidae_Actinopteri_XP_005473561.1/XP_019217465.1			
	Oryzias_latipes_(Japanese_medaka)_Adrianichthyidae_Actinopteri_XP_011477214.1			
	Otolemur_garnettii_(small-eared_galago)_Galagidae_Mammalia_XP_003784985.1			
	Ovis_aries_(sheep)_Bovidae_Mammalia_XP_012001778.1			
	Ovis_aries_musimon_(mouflon)_Bovidae_Mammalia_XP_011969055.1/XP_011975592.1/XP_012006067.1/XP_012006068.1			
	Pan_paniscus_(pygmy_chimpanzee)_Hominidae_Mammalia_XP_003818684.2			
	Pan_troglodytes_(chimpanzee)_Hominidae_Mammalia_XP_003309538.2/XP_009448027.1/XP_016792890.1			
	Panthera_pardus_(leopard)_Felidae_Mammalia_XP_019318144.1/XP_019318145.1			
	Panthera_tigris_altaica_(Amur_tiger)_Felidae_Mammalia_XP_015390390.1			
	Papio_anubis_(olive_baboon)_Cercopithecidae_Mammalia_XP_003908021.1/XP_003908023.1/XP_009181373.1/XP_009181375.1			
	Parasteatoda_tepidariorum_(common_house_spider)_Theridiidae_Arachnida_XP_021001893.1			
	Parus_major_(Great_Tit)_Paridae_Aves_XP_015483206.1/XP_015483207.1/XP_015490506.1/XP_015490507.1/XP_015497801.1/XP_015497802.1/XP_015497803.1/XP_018863084.1/XP_018863085.1			
	Phascolarctos_cinereus_(koala)_Phascolarctidae_Mammalia_XP_020858503.1/XP_020858504.1			
	Physeter_catodon_(sperm_whale)_Physeteridae_Mammalia_XP_007122524.1			
	Poecilia_latipinna_(sailfin_molly)_Poeciliidae_Actinopteri_XP_014889784.1			
	Pogona_vitticeps_(central_bearded_dragon)_Agamidae_XP_020643657.1			
	Pongo_abelii_(Sumatran_orangutan)_Hominidae_Mammalia_XP_002812918.2			
	Propithecus_coquereli_(Coquerel's_sifaka)_Indriidae_Mammalia_XP_012498227.1			
	Pseudopodoces_humilis_(Tibetan_ground-tit)_Paridae_Aves_XP_005519768.1/XP_014116676.1/XP_014116677.1/XP_014116994.1			
	Pteropus_alecto_(black_flying_fox)_Pteropodidae_Mammalia_ELK03988.1/ELK17190.1/XP_015440938.1/XP_015453665.1			
	Pteropus_vampyrus_(large_flying_fox)_Pteropodidae_Mammalia_XP_011356870.1			
	Pygocentrus_nattereri_(red-bellied_piranha)_Serrasalmidae_Actinopteri_XP_017575421.1			
	Rattus_norvegicus_(Norway_rat)_Muridae_Mammalia_NP_001256967.1/XP_006235131.1			
	Rhinolophus_sinicus_(Chinese_rufous_horseshoe_bat)_Rhinolophidae_Mammalia_XP_019597384.1/XP_019606349.1			
	Rhinopithecus_bieti_(black_snub-nosed_monkey)_Cercopithecidae_Mammalia_XP_017712261.1			
	Rhinopithecus_roxellana_(golden_snub-nosed_monkey)_Cercopithecidae_Mammalia_XP_010377244.1			
	Rousettus_aegyptiacus_(Egyptian_rousette)_Pteropodidae_Mammalia_XP_015992371.1			
	Saimiri_boliviensis_boliviensis_(Bolivian_squirrel_monkey)_Cebidae_Mammalia_XP_010334603.1/XP_010342779.1/XP_010342783.1			
	Salmo_salar_(Atlantic_salmon)_Salmonidae_Actinopteri_XP_014016984.1			
	Scleropages_formosus_(Asian_bonytongue)_Osteoglossidae_Actinopteri_KPP73653.1			
	Serinus_canaria_(common_canary)_Fringillidae_Aves_XP_018765601.1			
	Strongylocentrotus_purpuratus_(purple_sea_urchin)_Strongylocentrotidae_Echinoidea_XP_011682282.1			
	Takifugu_rubripes_(torafugu)_Tetraodontidae_Actinopteri_XP_003979325.1/XP_011613797.1			
	Trichechus_manatus_latirostris_(Florida_manatee)_Trichechidae_Mammalia_XP_004376385.1/XP_004381113.1			
	Ursus_maritimus_(polar_bear)_Ursidae_Mammalia_XP_008705822.1			
	Varroa_destructor_(honeybee_mite)_Varroidae_Arachnida_XP_022645397.1/XP_022645398.1/XP_022653397.1/XP_022653398.1			
	Varroa_jacobsoni_(mites_&_ticks)_Varroidae_Arachnida_XP_022696845.1/XP_022696846.1/XP_022699352.1/XP_022699362.1			
	Vicugna_pacos_(alpaca)_Camelidae_Mammalia_XP_015102398.1			
	Xenopus_laevis_(African_clawed_frog)_Pipidae_Amphibia_XP_018096356.1/XP_018106832.1/XP_018108911.1			
	Xenopus_tropicalis_(tropical_clawed_frog)_Pipidae_Amphibia_OCA23951.1/OCA42244.1			
	Zonotrichia_albicollis_(white-throated_sparrow)_Passerellidae_Aves_XP_014127291.1			

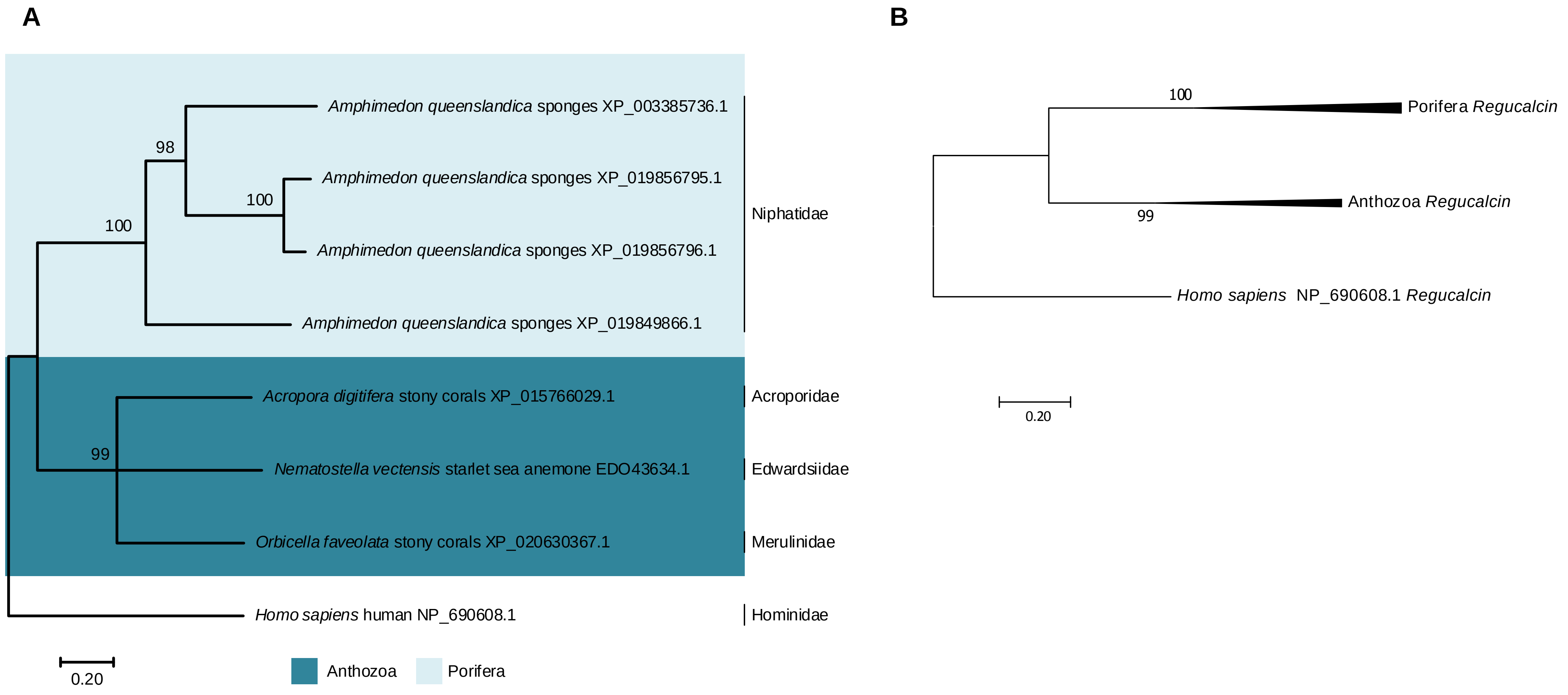

Additional file 1: Fig. S1

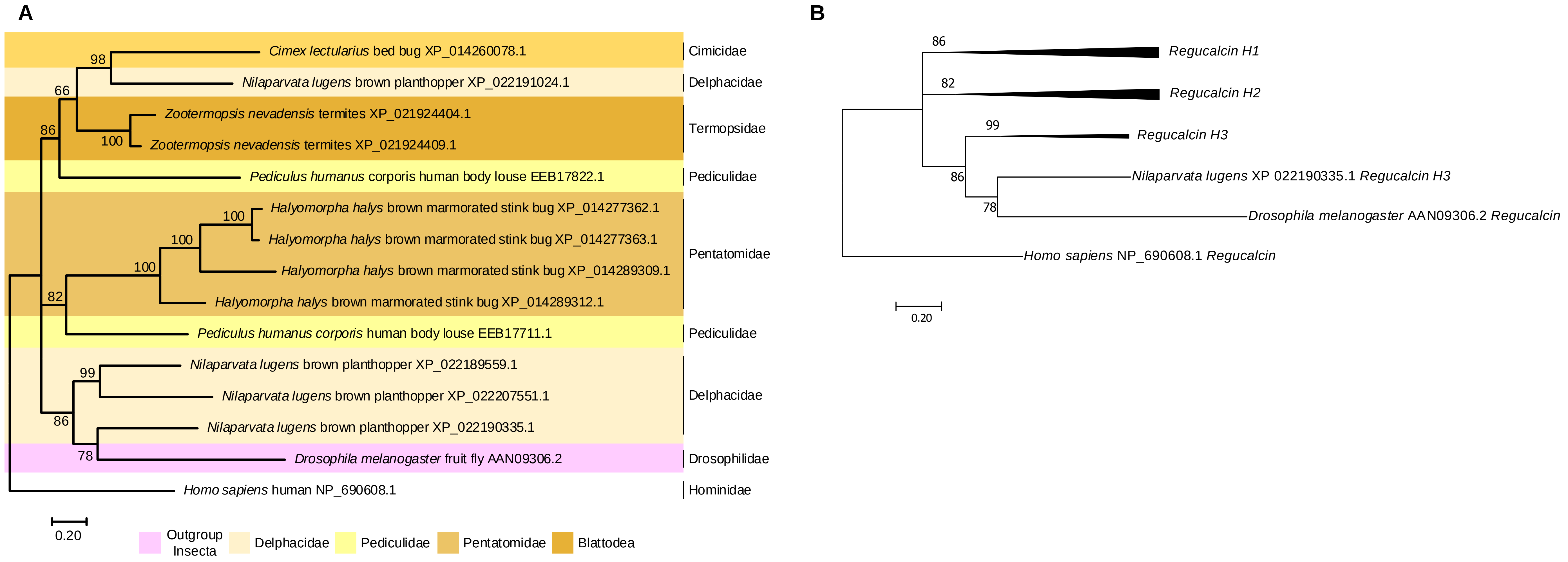

Additional file 1: Fig. S2

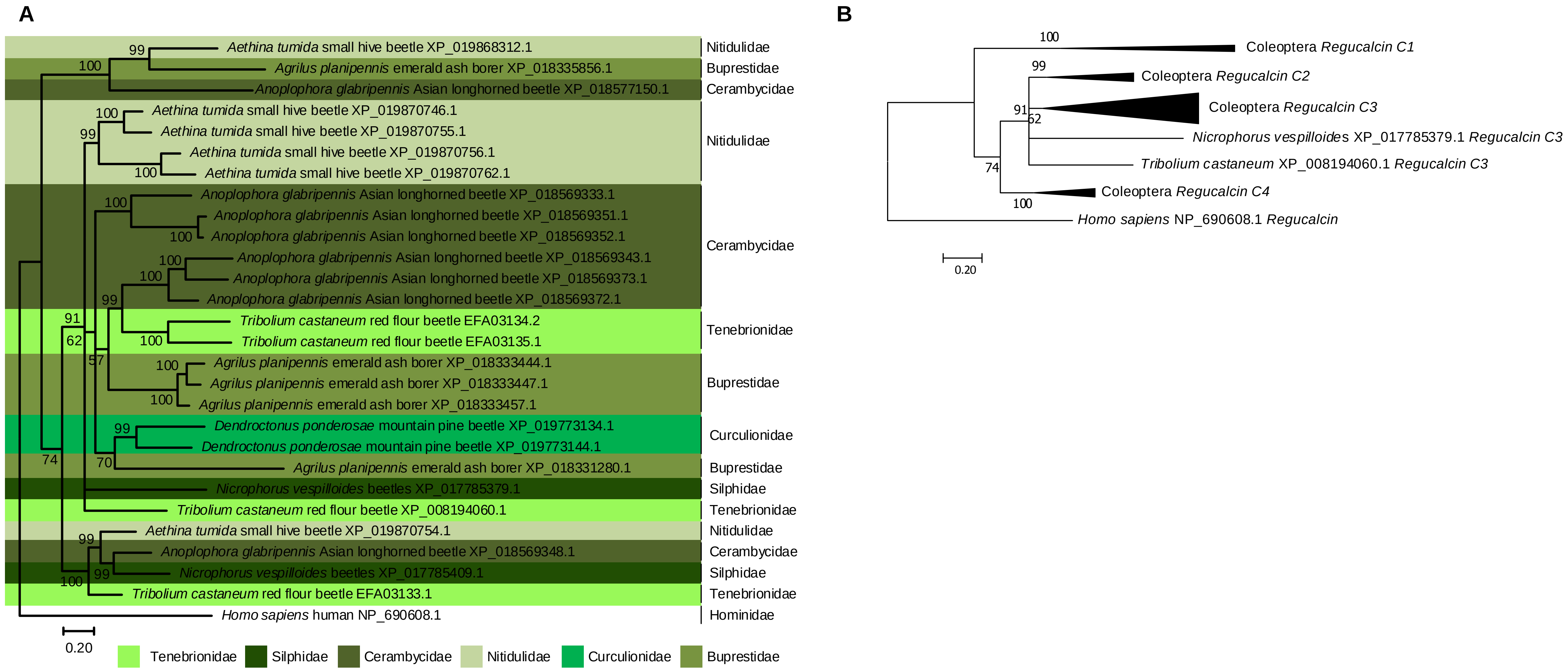

Additional file 1: Fig. S3

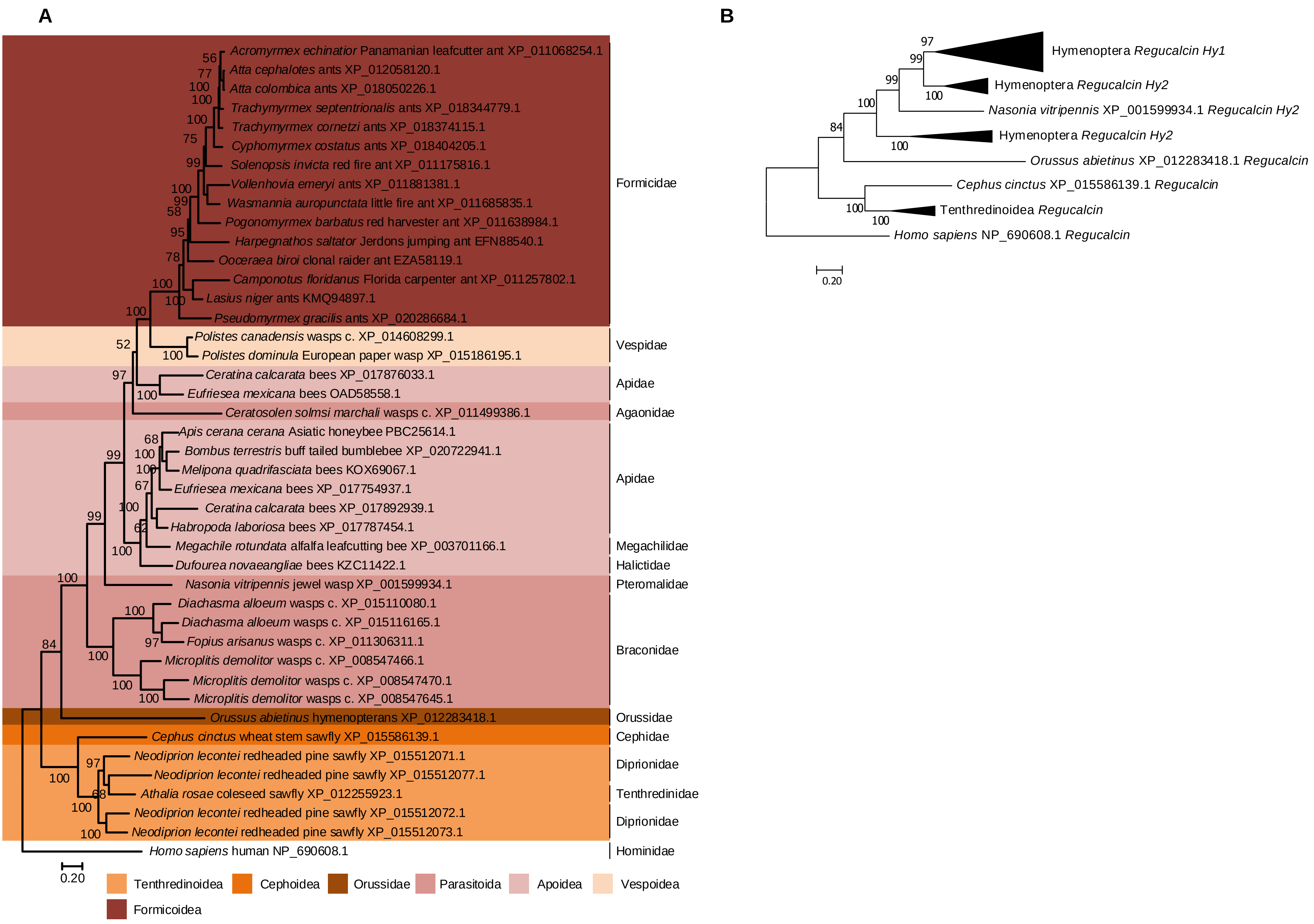

Additional file 1: Fig. S4

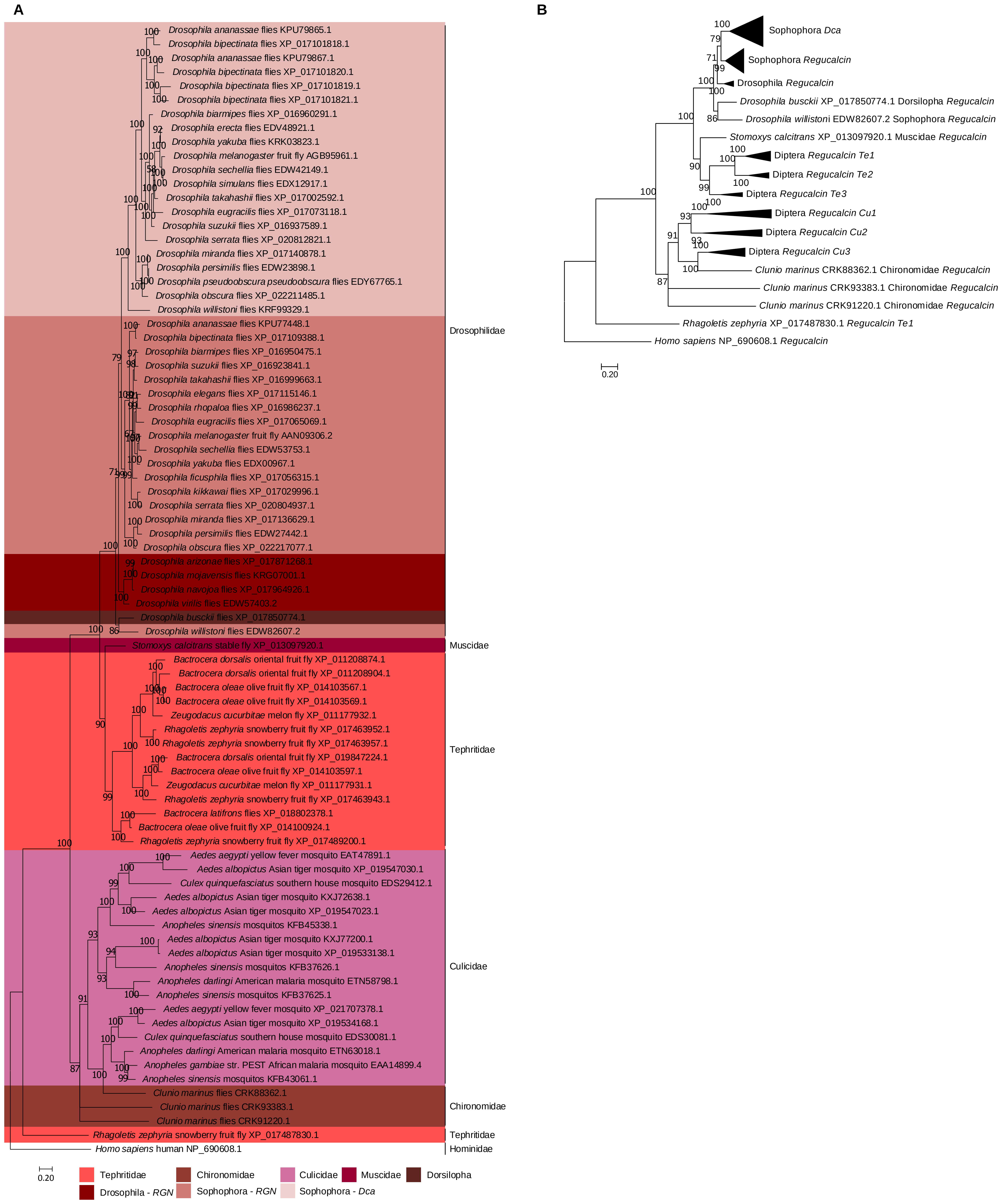

Additional file 1: Fig. S5

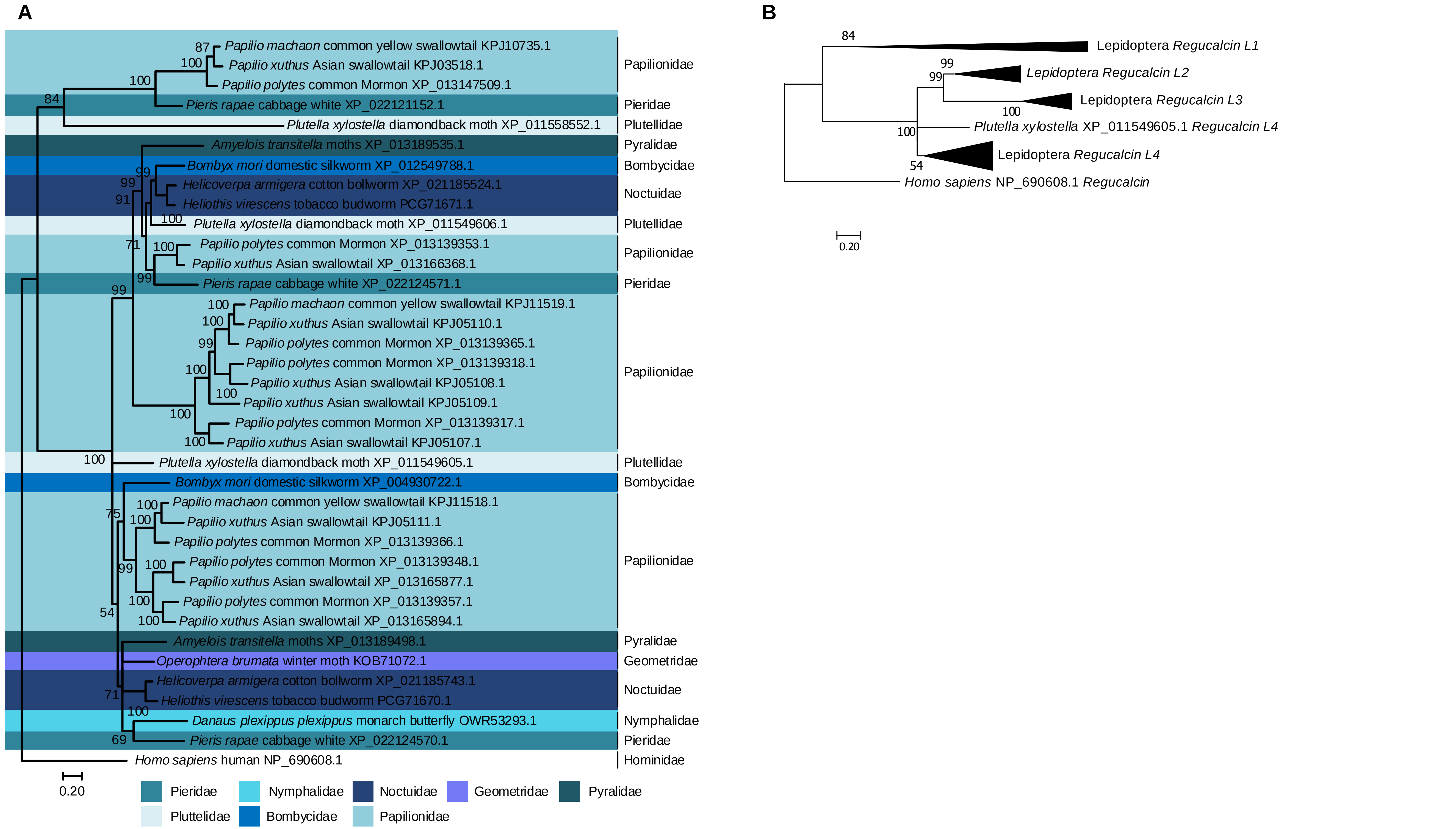

Additional file 1: Fig. S6

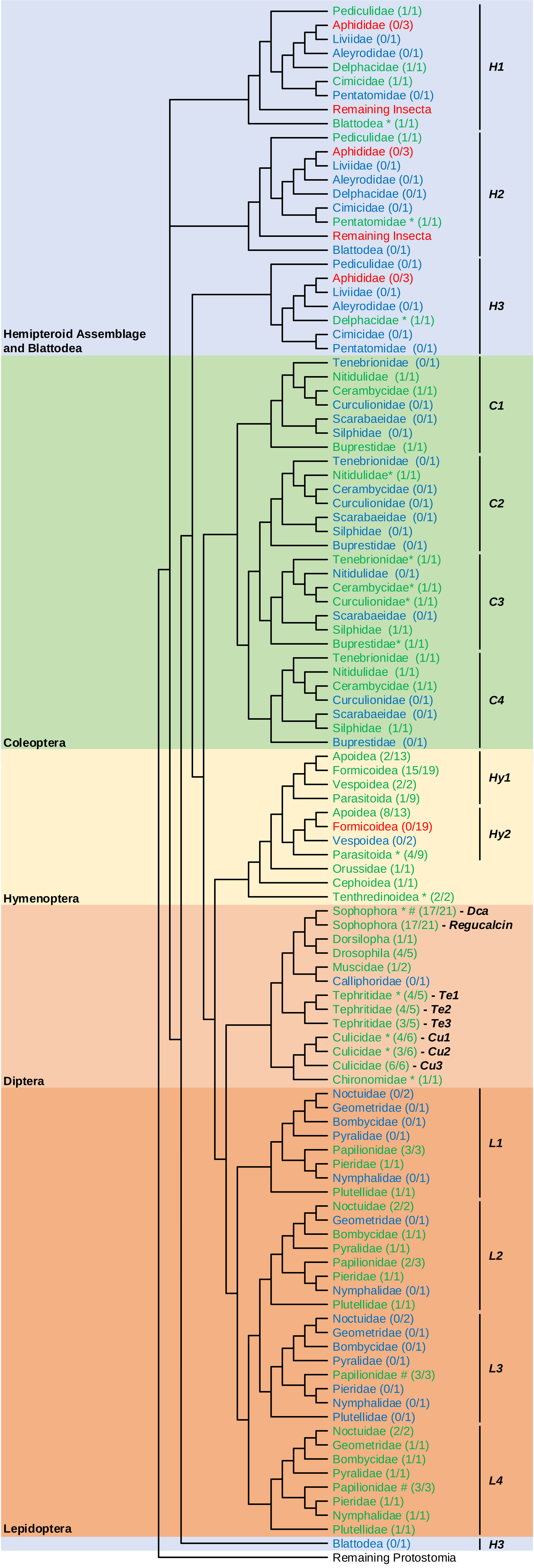

Additional file 1: Fig. S7

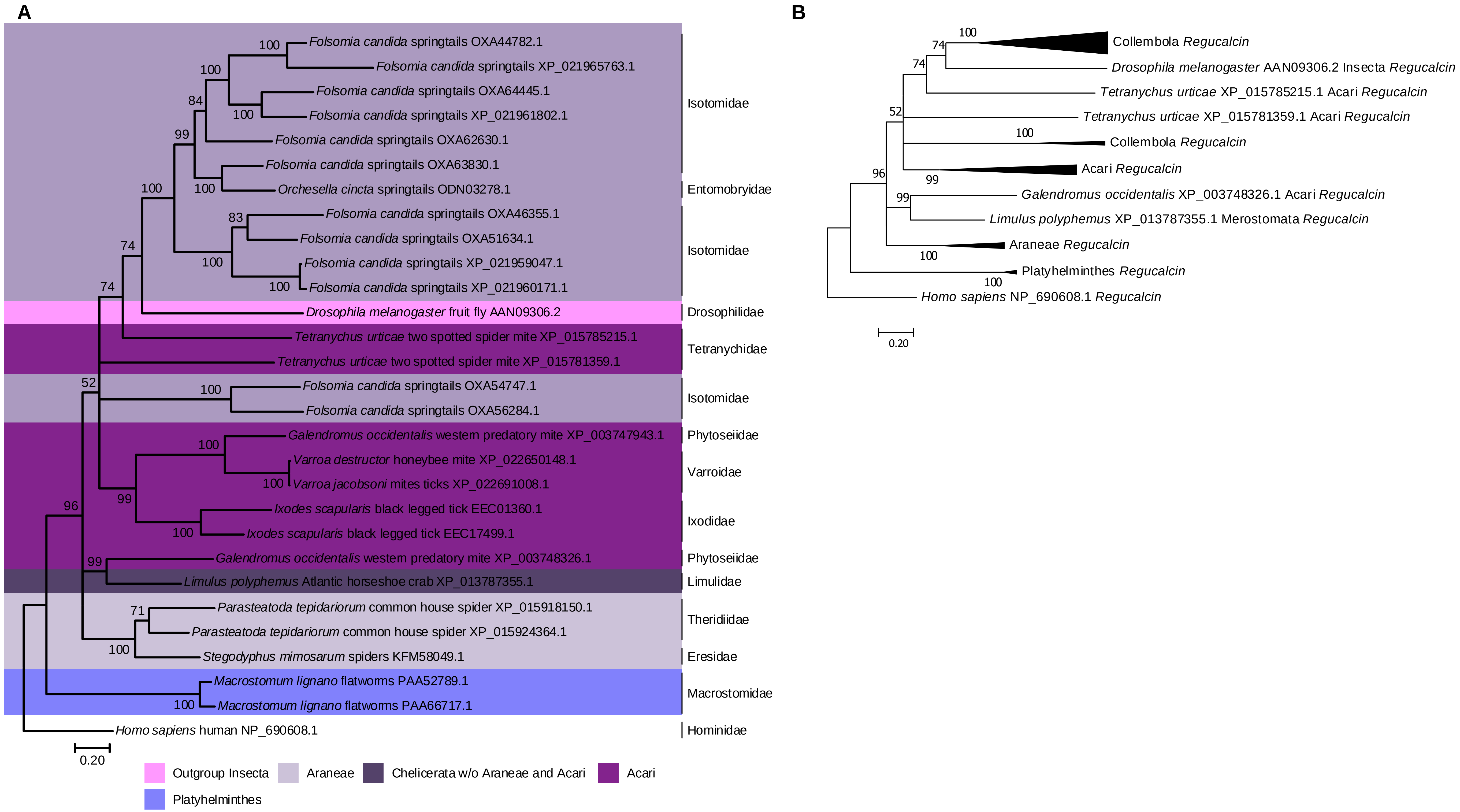

Additional file 1: Fig. S8

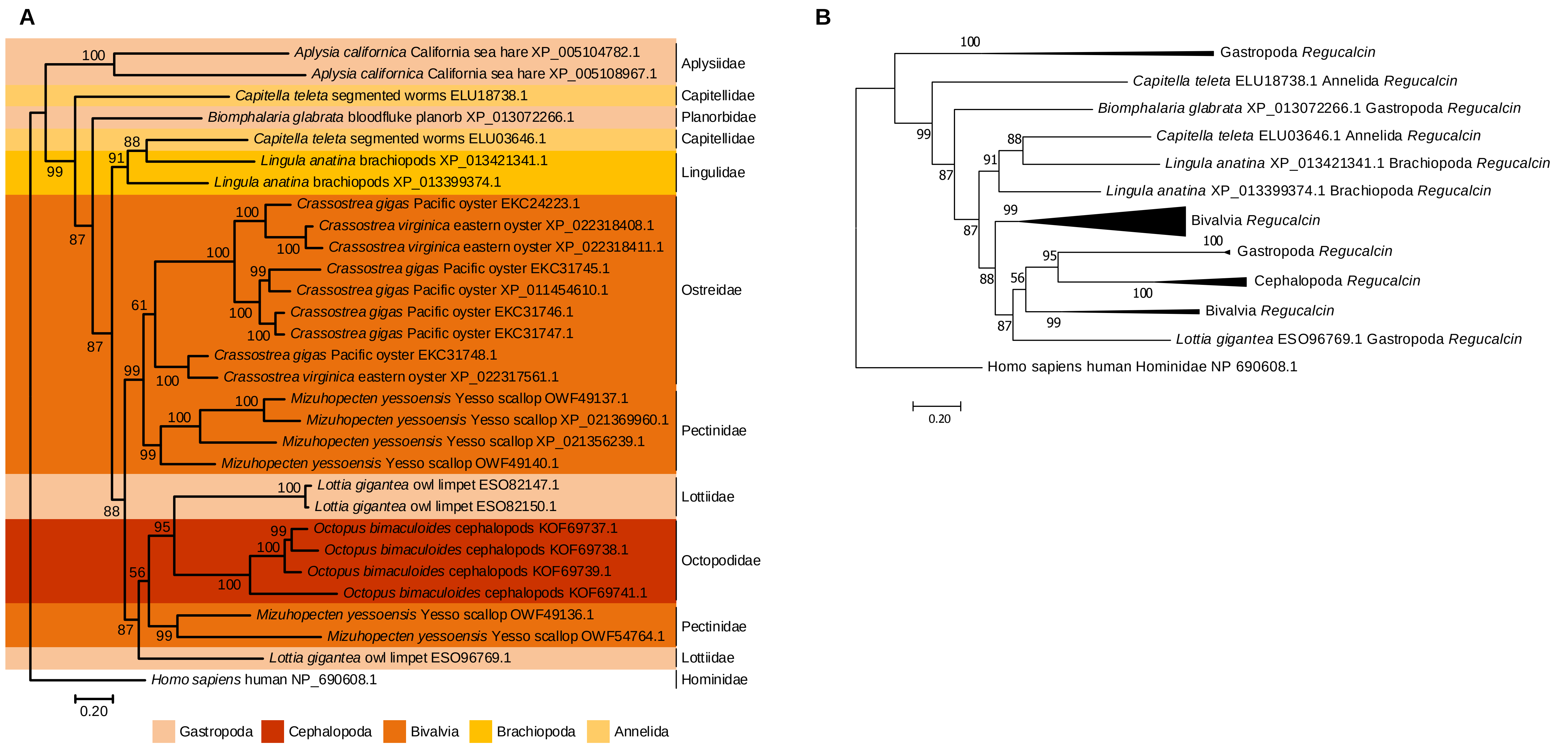

Additional file 1: Fig. S9

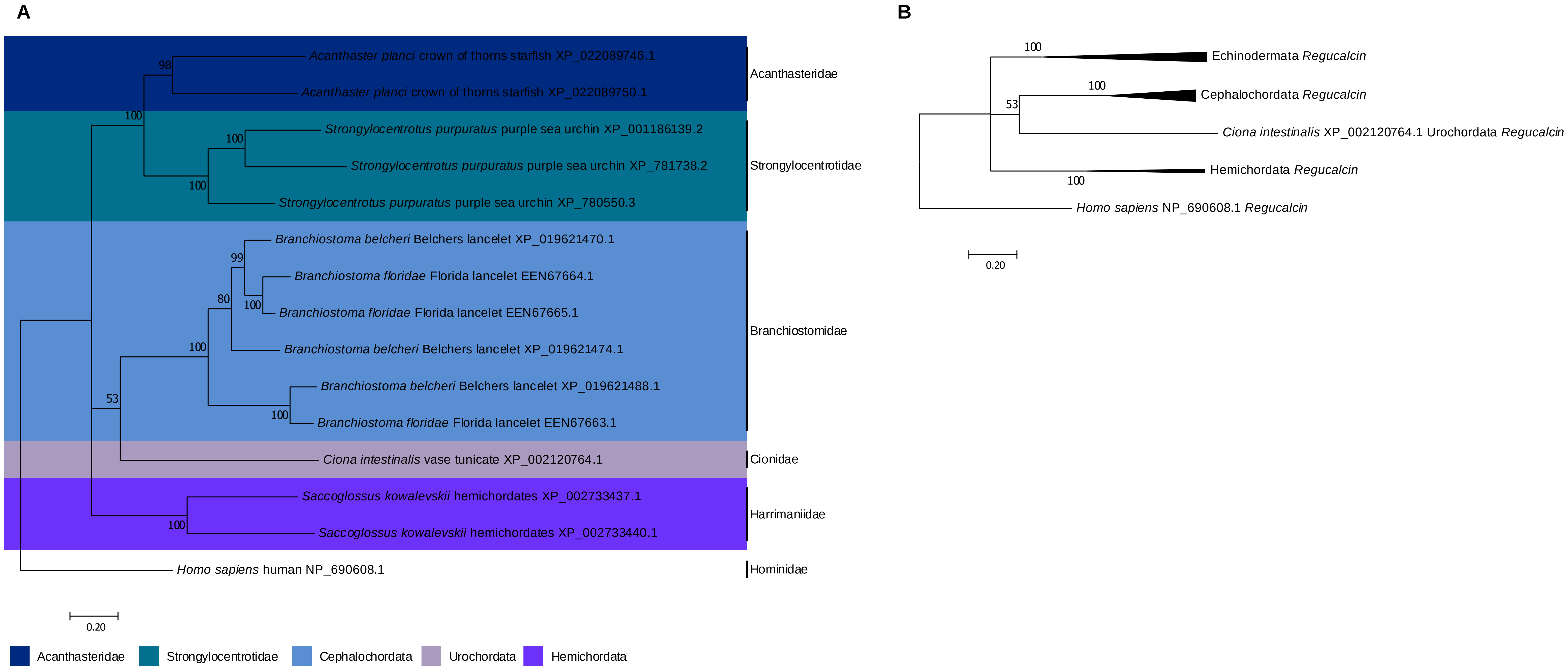

Additional file 1: Fig. S10

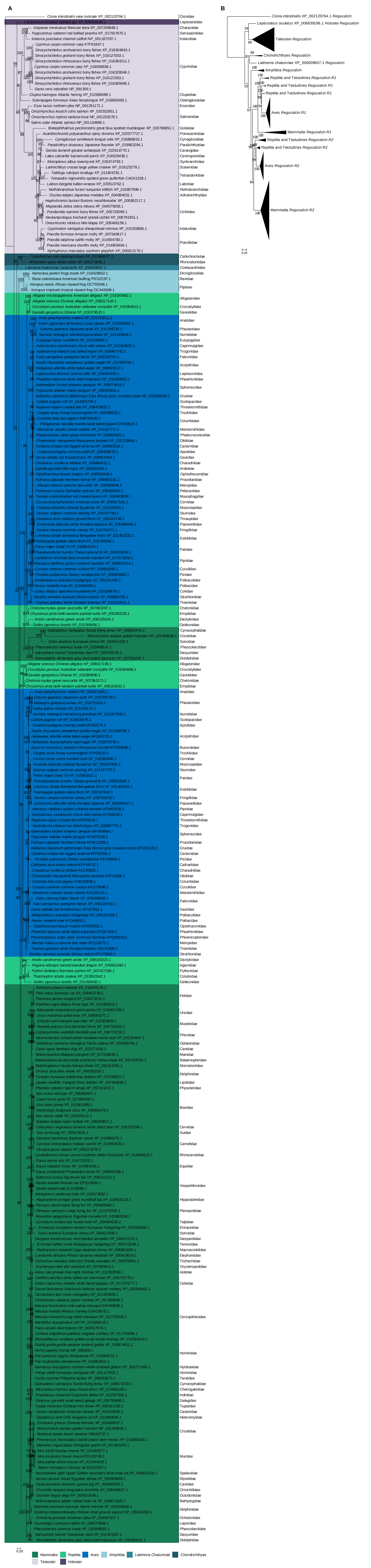

Additional file 1: Fig. S11

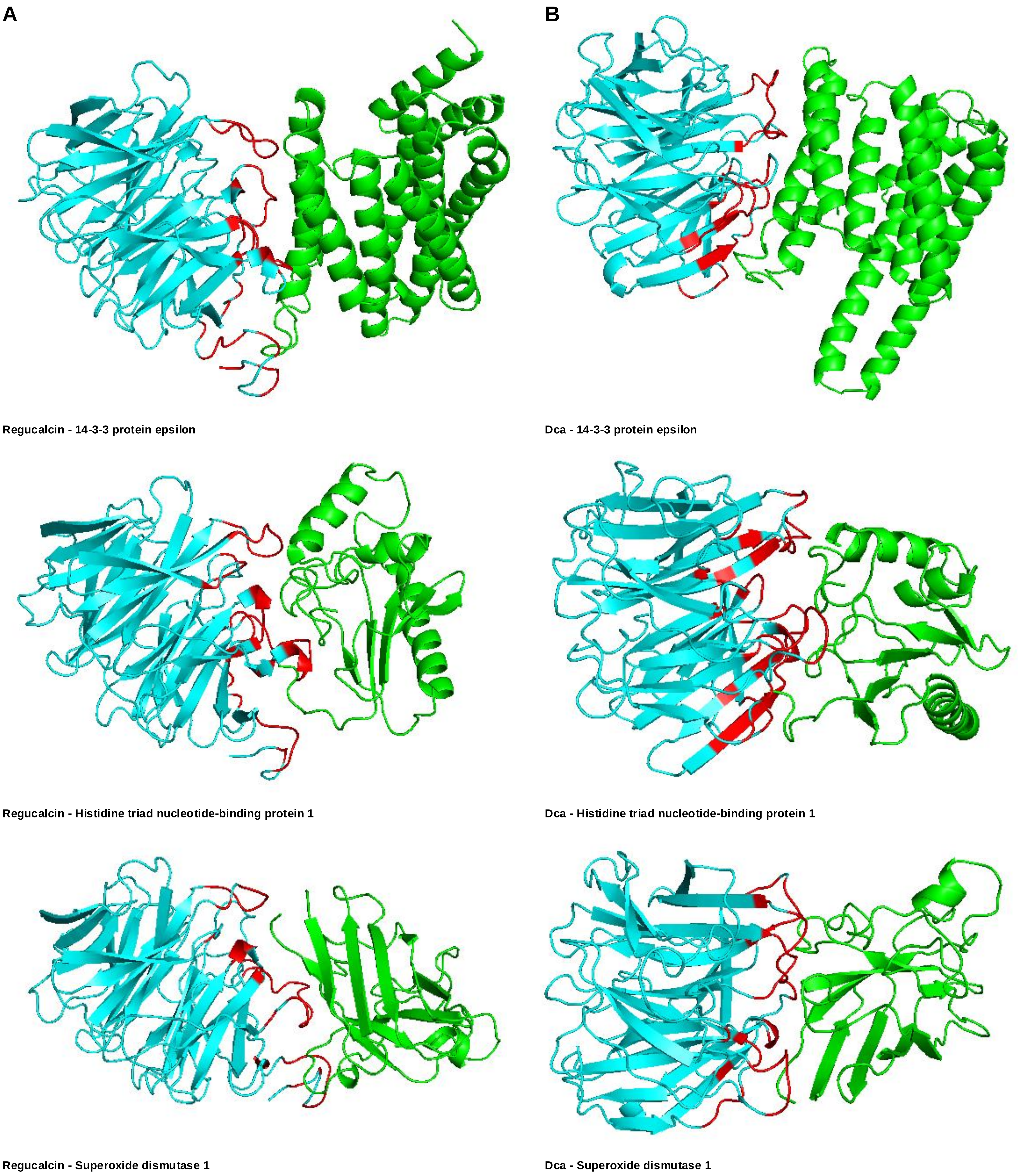

Additional file 1: Fig. S12

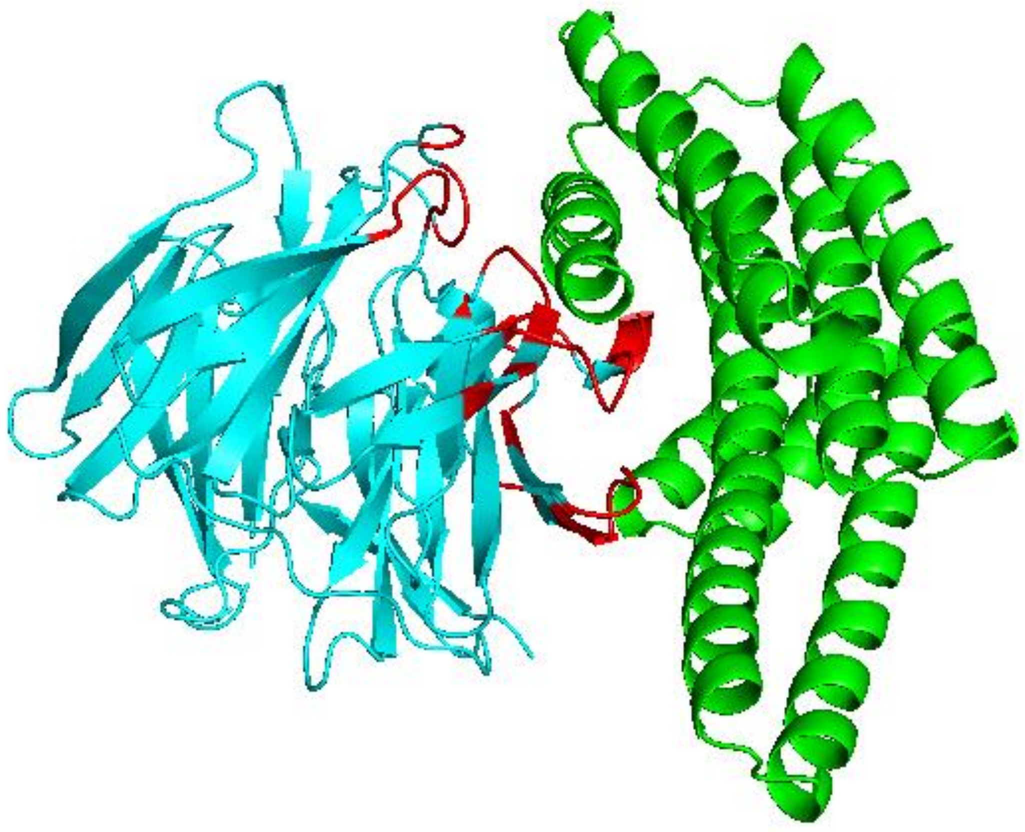

Regucalcin - 14-3-3 protein epsilon (Human)

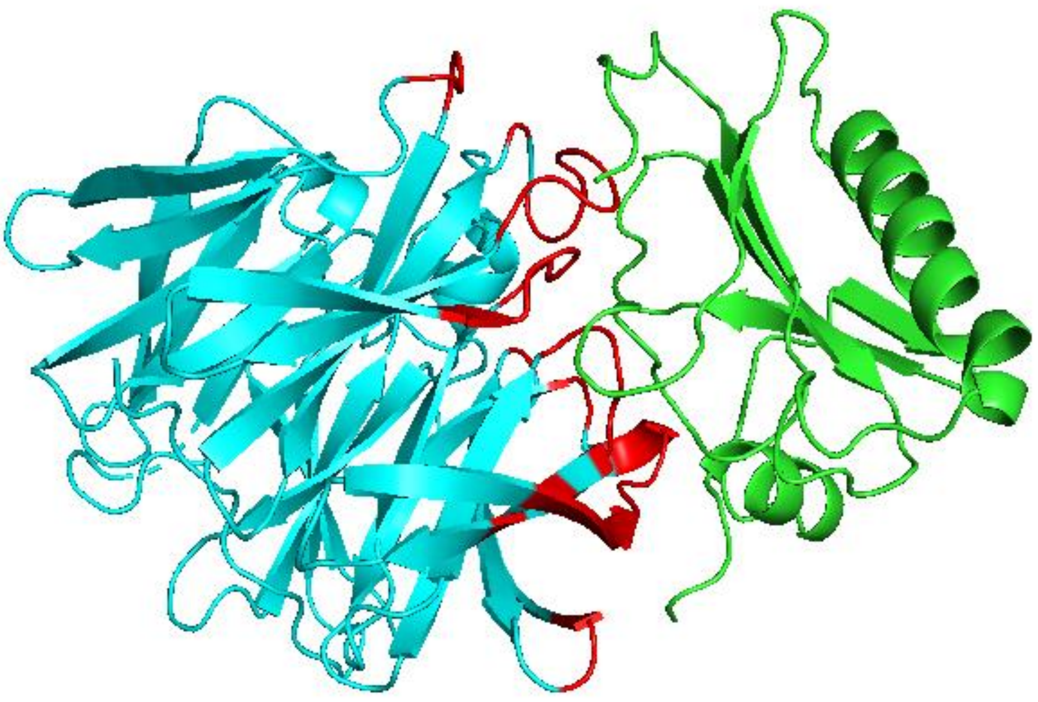

Regucalcin - Histidine triad nucleotide-binding protein 1 (Human)

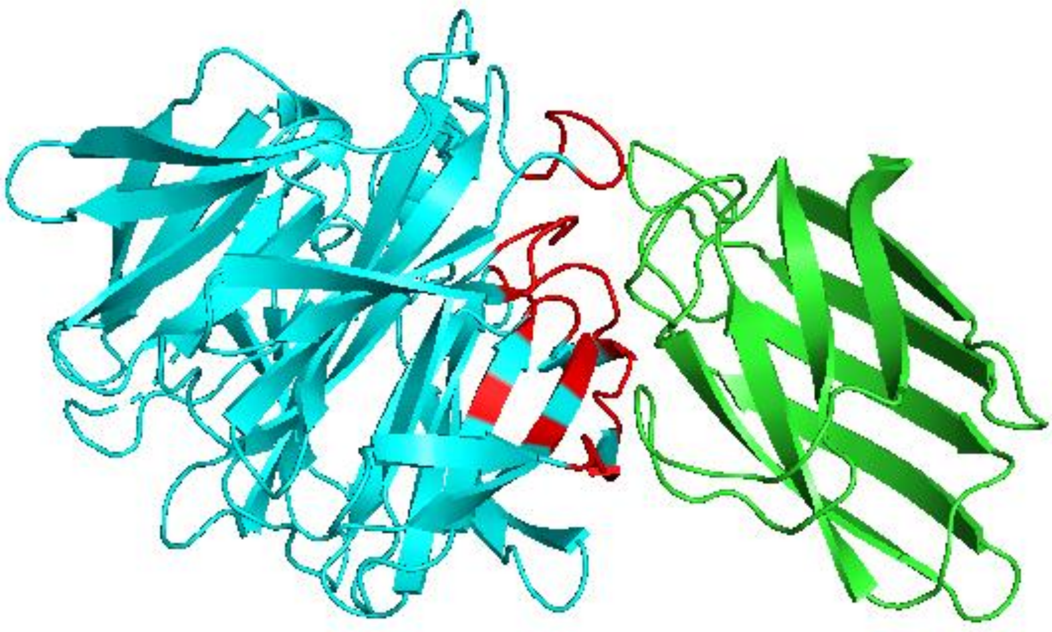

Regucalcin - Superoxide dismutase 1 (Human)

Additional file 1: Fig. S13

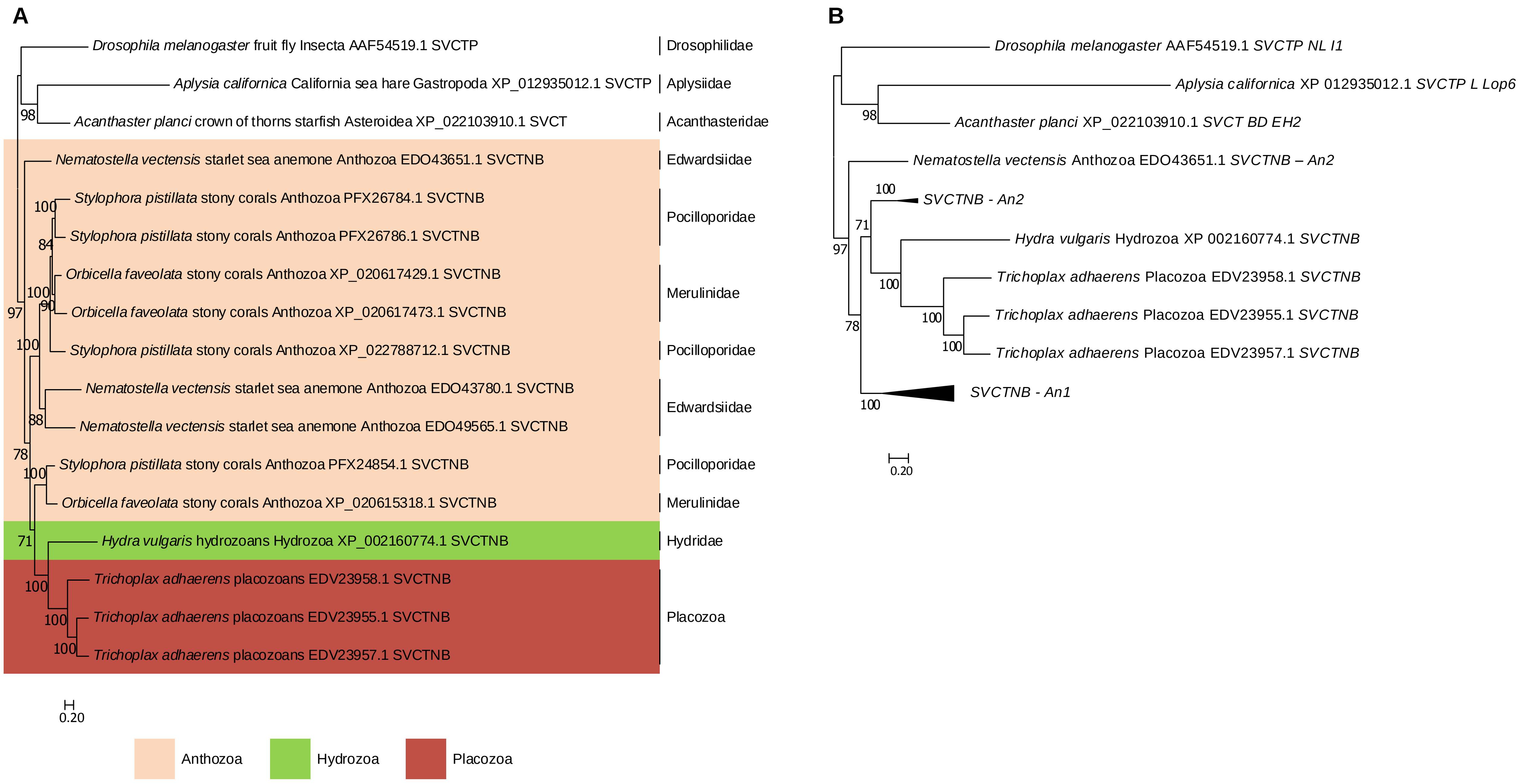

Additional file 1: Fig. S14

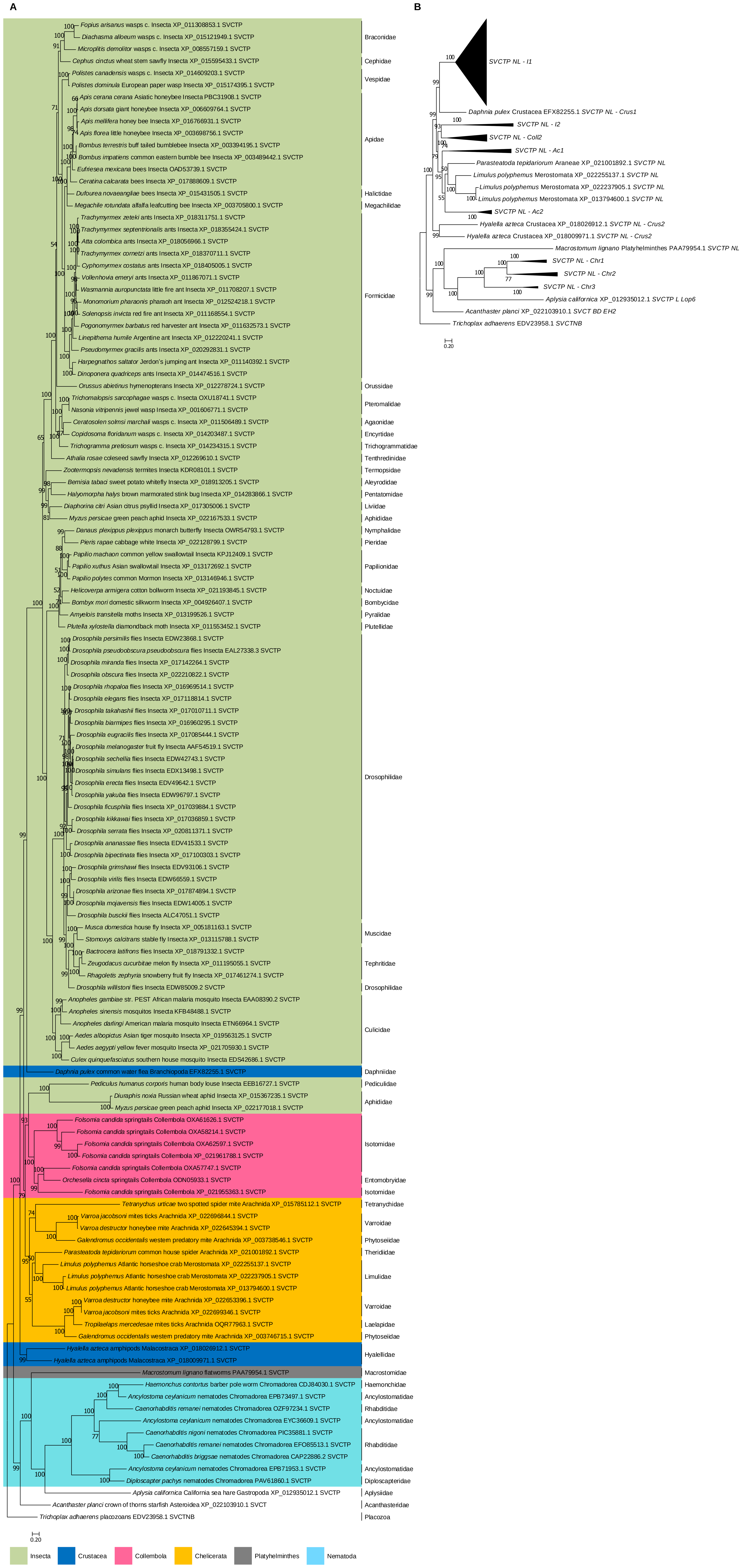

Additional file 1: Fig. S15

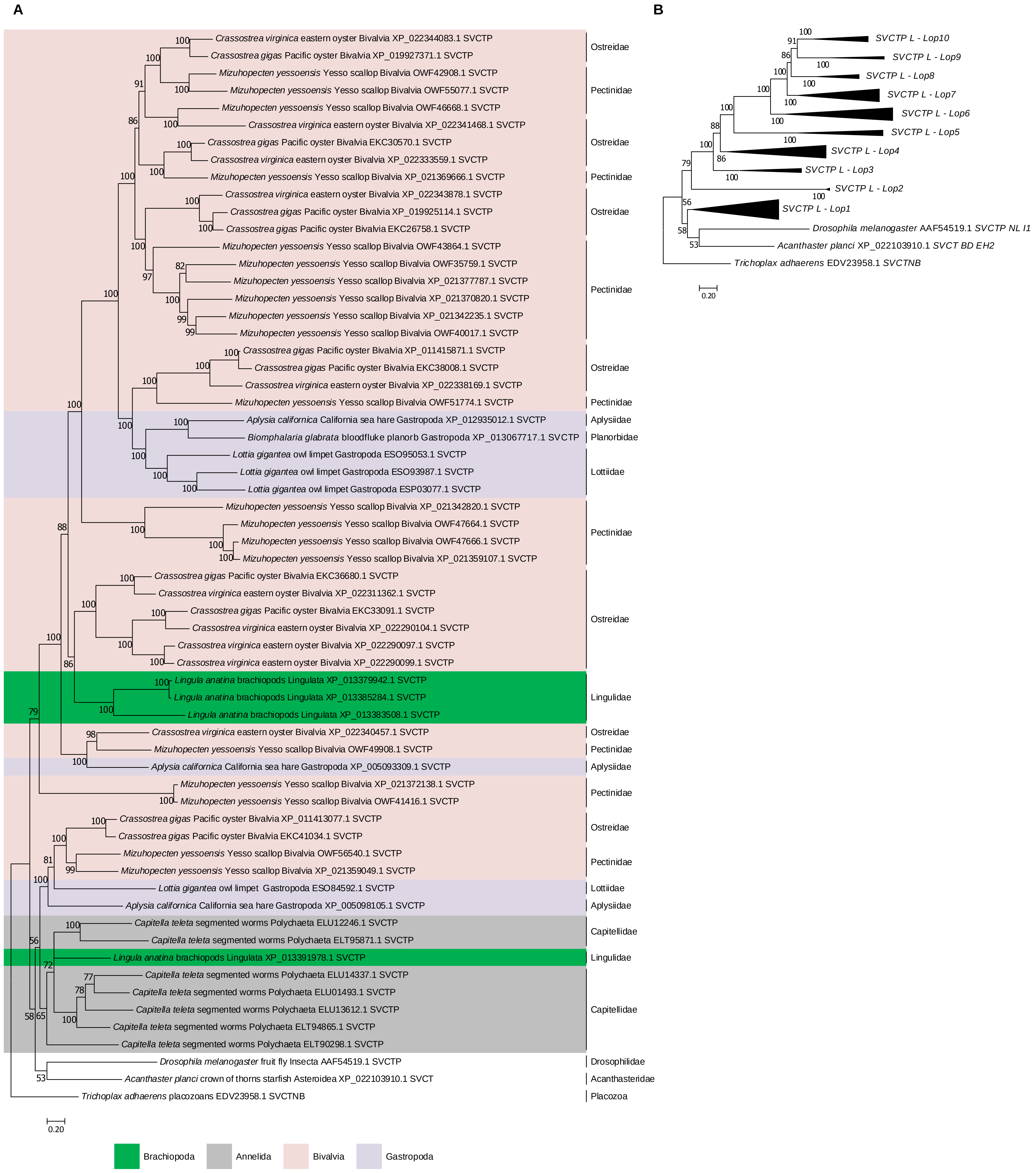

Additional file 1: Fig. S16

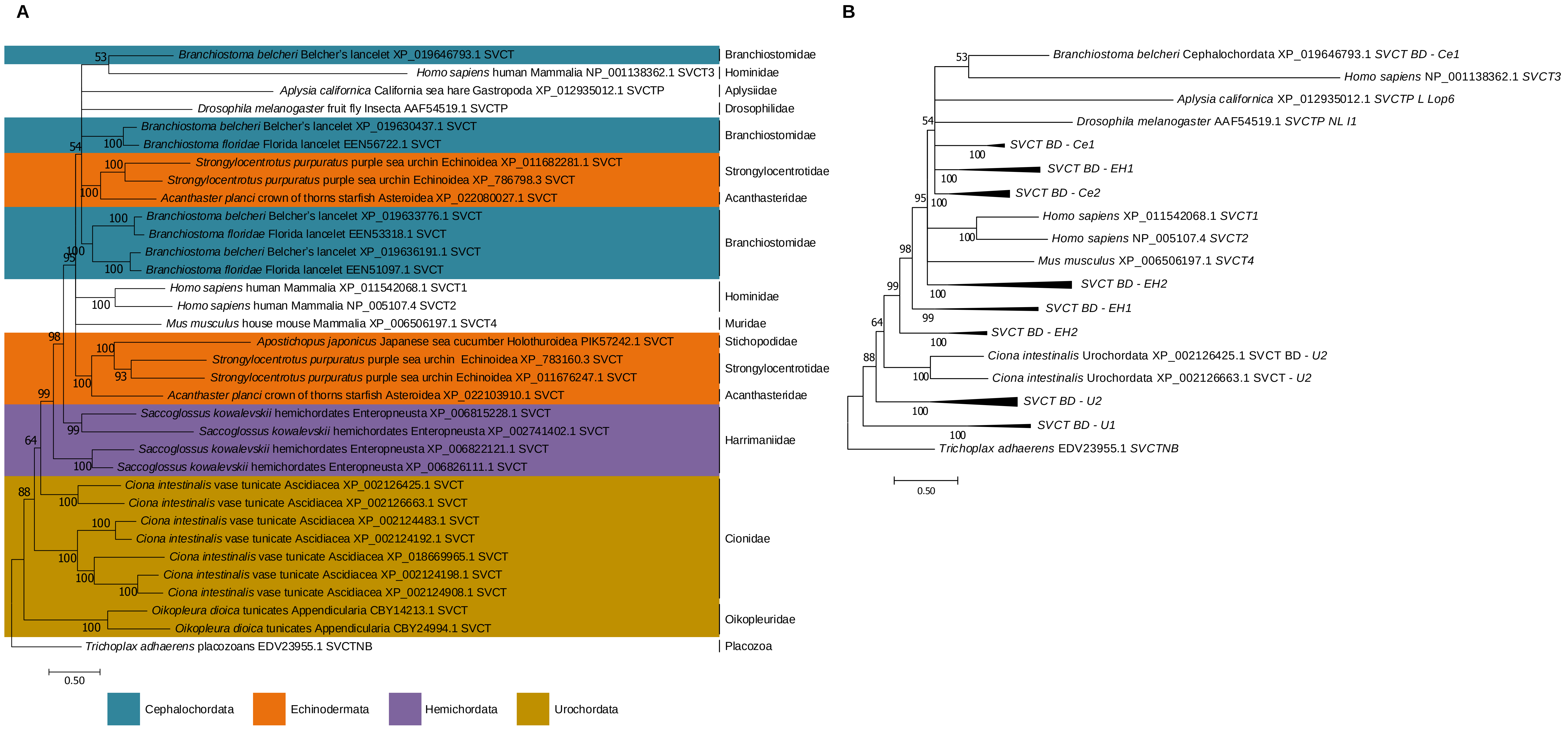

Additional file 1: Fig. S17

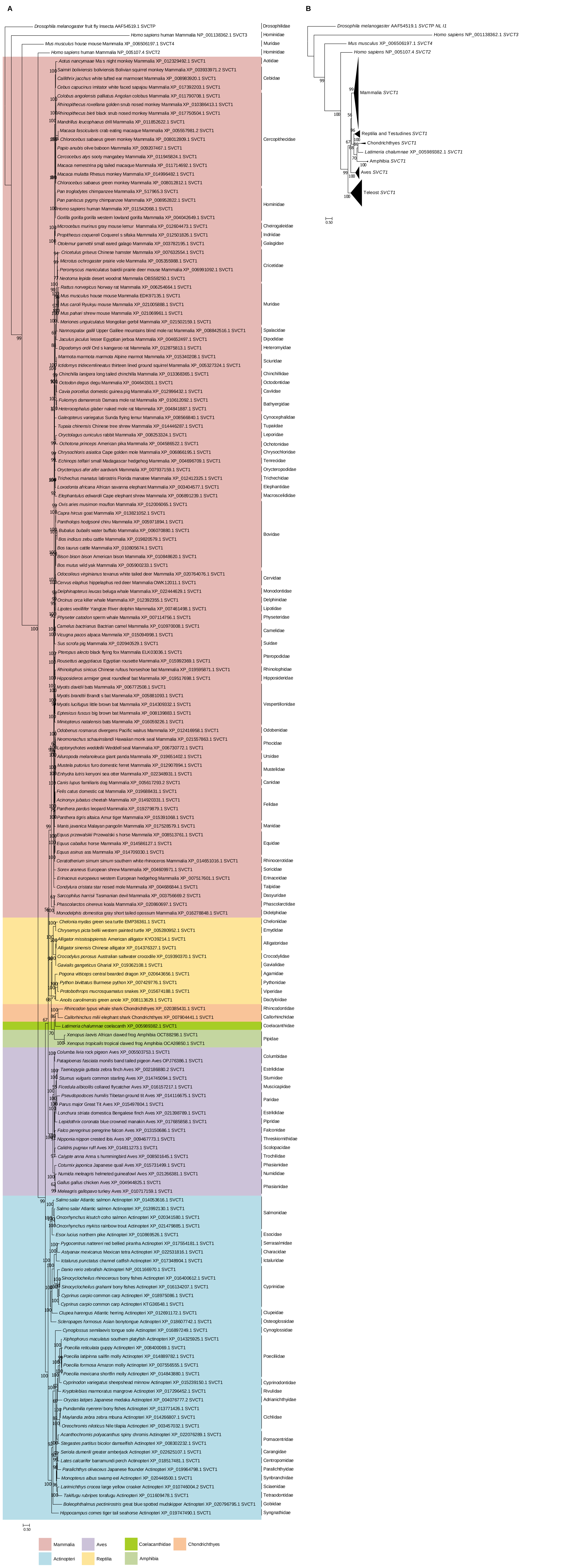

Additional file 1: Fig. S18

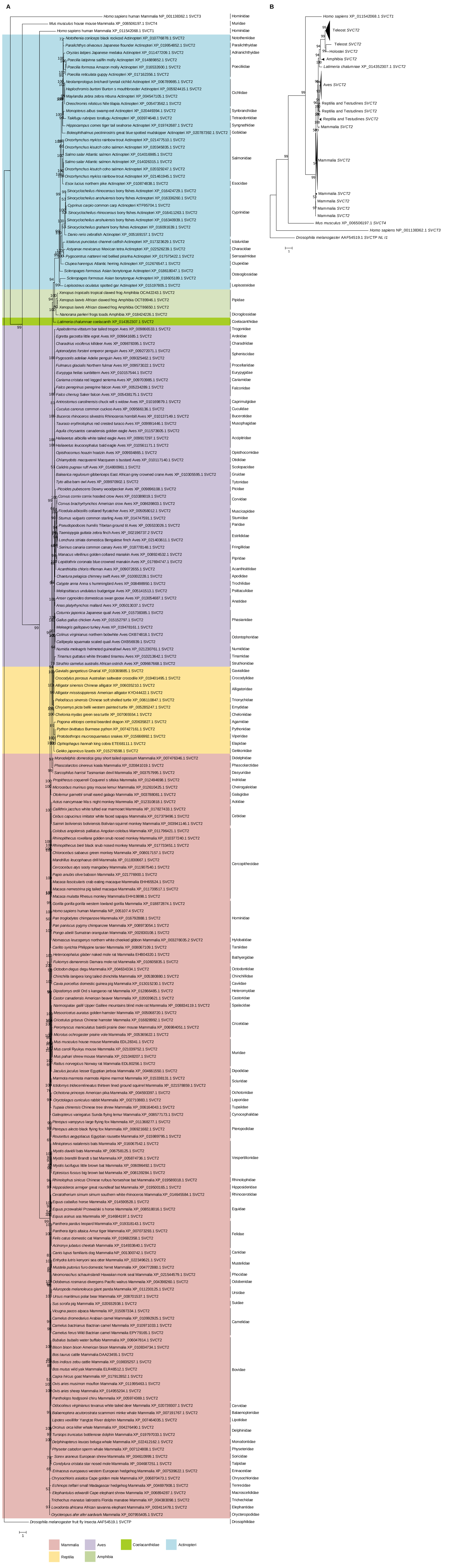

Additional file 1: Fig. S19

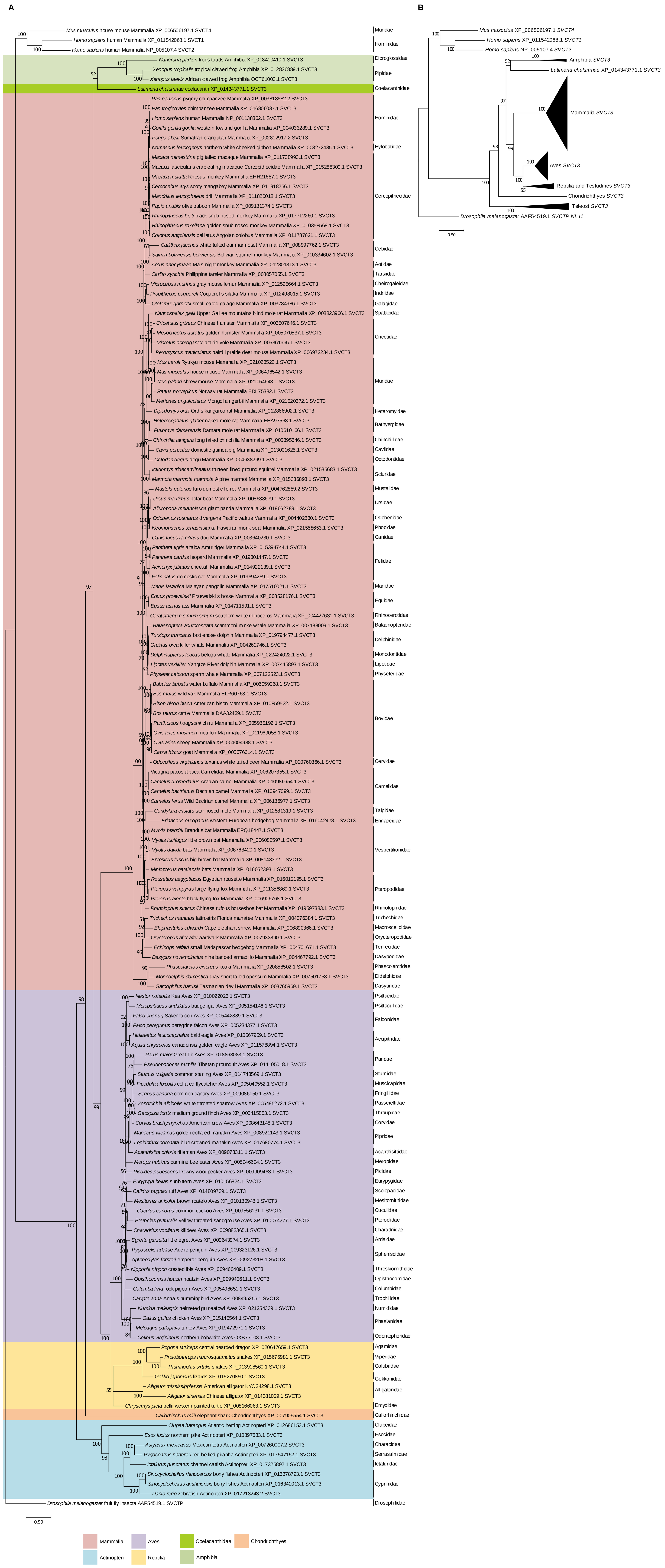

Additional file 1: Fig. S20

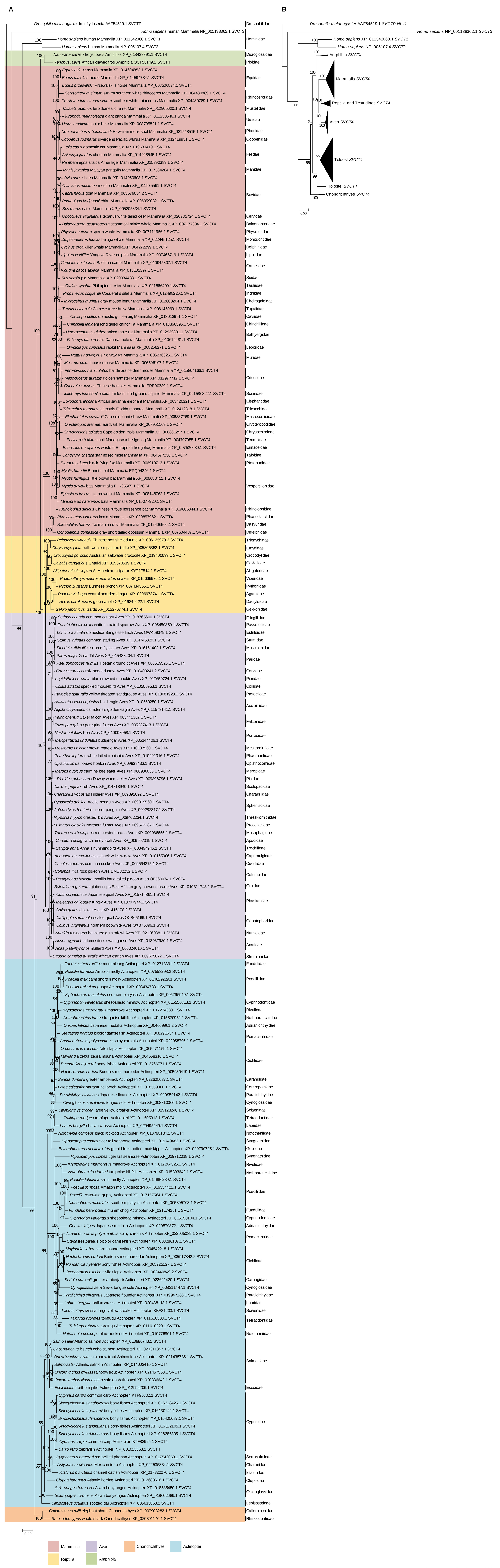

Additional file 1: Fig. S21

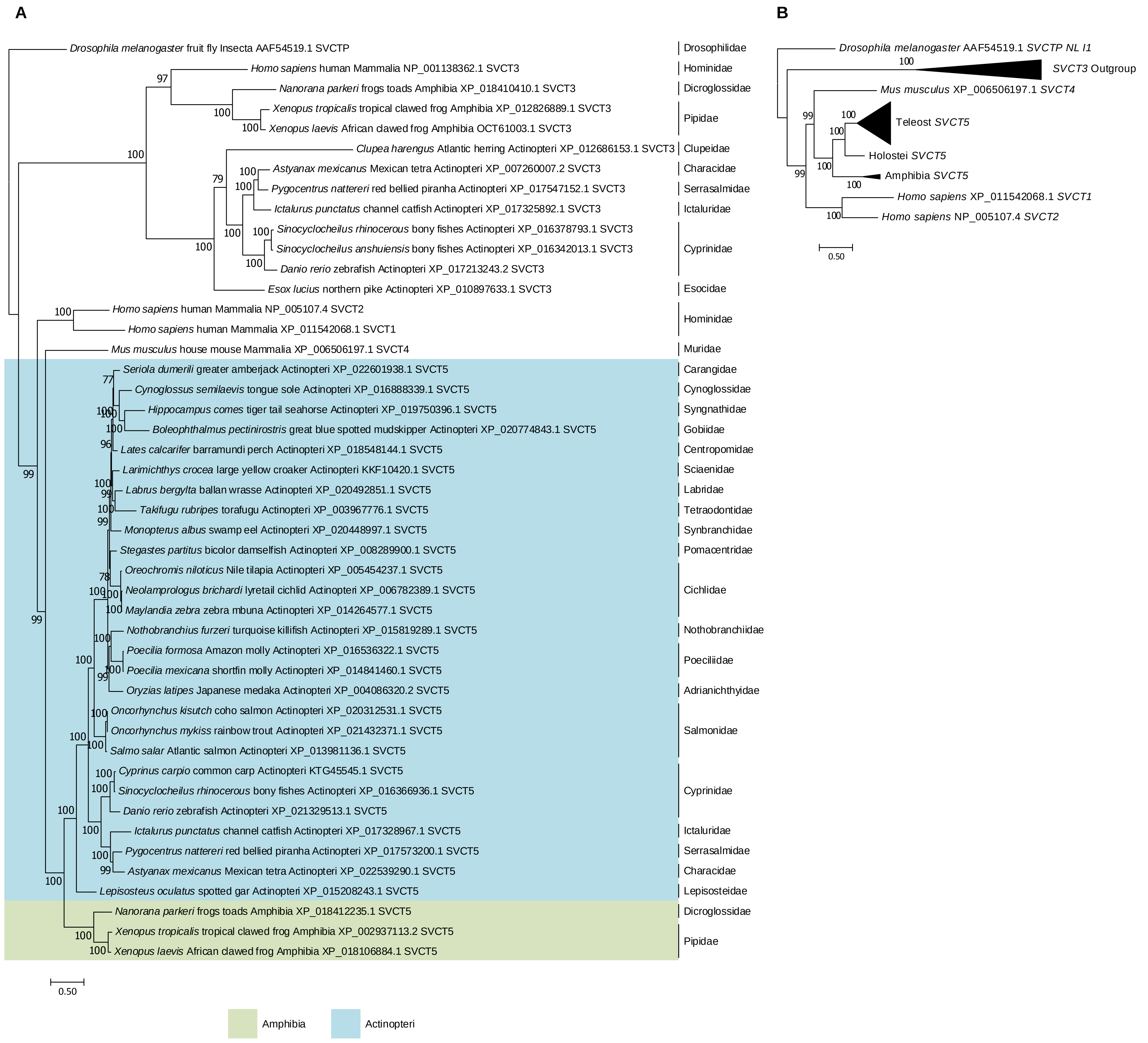

Additional file 1: Fig. S22
